# On-demand ferrofluid droplet formation with non-linear magnetic permeability in the presence of high non-uniform magnetic fields

**DOI:** 10.1038/s41598-022-14624-w

**Published:** 2022-06-27

**Authors:** Mohamad Ali Bijarchi, Mohammad Yaghoobi, Amirhossein Favakeh, Mohammad Behshad Shafii

**Affiliations:** 1 Department of Mechanical Engineering, Sharif University of Technology, Tehran, Iran; 2 Sharif Energy, Water and Environment Institute (SEWEI), Tehran, Iran

**Keywords:** Engineering, Mechanical engineering, Fluid dynamics

## Abstract

The magnetic actuation of ferrofluid droplets offers an inspiring tool in widespread engineering and biological applications. In this study, the dynamics of ferrofluid droplet generation with a Drop-on-Demand feature under a non-uniform magnetic field is investigated by multiscale numerical modeling. Langevin equation is assumed for ferrofluid magnetic susceptibility due to the strong applied magnetic field. Large and small computational domains are considered. In the larger domain, the magnetic field is obtained by solving Maxwell equations. In the smaller domain, a coupling of continuity, Navier Stokes, two-phase flow, and Maxwell equations are solved by utilizing the magnetic field achieved by the larger domain for the boundary condition. The Finite volume method and coupling of level-set and Volume of Fluid methods are used for solving equations. The droplet formation is simulated in a two-dimensional axisymmetric domain. The method of solving fluid and magnetic equations is validated using a benchmark. Then, ferrofluid droplet formation is investigated experimentally, and the numerical results showed good agreement with the experimental data. The effect of 12 dimensionless parameters, including the ratio of magnetic, gravitational, and surface tension forces, the ratio of the nozzle and magnetic coil dimensions, and ferrofluid to continuous-phase properties ratios are studied. The results showed that by increasing the magnetic Bond number, gravitational Bond number, Ohnesorge number, dimensionless saturation magnetization, initial magnetic susceptibility of ferrofluid, the generated droplet diameter reduces, whereas the formation frequency increases. The same results were observed when decreasing the ferrite core diameter to outer nozzle diameter, density, and viscosity ratios.

## Introduction

Many studies have focused on droplet deformation, breakup, and generation for about a century due to their widespread applications and rich underlying physics. The dynamics of droplet formation has been investigated numerically, experimentally, and theoretically in different geometries, including nozzles^1^ and lab-on-a-chip systems^2^. Droplet generation has been utilized in various applications, such as 3D printing^3^, electrosprays^4^, and bioengineering^5^. Droplet formation is performed by passive^6^ or active^7^ methods, which differ in the absence or presence of an external force. Electrical^8^, magnetic^9^, and acoustic^10^ forces have been the most external stimuli in the active methods.

Among different active methods, magnetic field actuation offers an inspiring and promising tool that provides wireless control of droplets, independent of their temperature and ion concentration^11^. Ferrofluid droplets are usually used in magnetic excitation. Ferrofluids are colloidal suspensions of magnetic nanoparticles (usually Fe_3_O_4_) with a diameter of less than 10 nm in a carrier fluid which can be oil or water^12^. Surfactants are coated around the nanoparticles to increase the stability of the suspension. Ferrofluids simultaneously have the magnetic properties of nanoparticles and the fluidity of carrier fluid. They have found various applications such as heat transfer enhancement^13,14^, mixing^15^, droplet generation from the nozzles and microfluidics^16,17^, droplet splitting^18,19^, merging^20^, and manipulation in magnetic digital microfluidics^21,22^. Mefford et al.^23^ showed the application of utilizing ferrofluid droplets for restorative treatment of retinal detachment by investigating the ferrofluid droplet movement in a viscous medium using a small magnet. Fan et al.^24^ showed on-demand merging and splitting multiple functionalities of ferrofluid droplets as soft robots in magnetic digital microfluidics.

Numerical methods have been utilized for simulating the ferrofluid droplet deformation and generation since the branch of ferrohydrodynamics introduced by Rosenweig^25^. Researchers have numerically studied the deformation of a ferrofluid droplet under external flows and uniform or non-uniform magnetic fields. Afkhami et al.^26^ investigated the effects of magnetic fields on deformations of a biocompatible ferrofluid droplet immersed in a highly viscous material. They focused their numerical simulations on an axisymmetric droplet under a uniform magnetic field. Their results strongly suggest that magnetic particle arrangement at the surface varies at different magnetic fields leading to a change in the surface tension. The simultaneous effect of applying magnetic and shear forces on ferrofluid droplets attracted researchers’ attention in recent years^27–30^. The droplet breakup and deformation have been investigated in these studies considering the balance between the magnetic and shear forces by capillary and magnetic Bond numbers. Afkhami et al.^31^ investigated droplet deformation and velocity in non-uniform magnetic fields. In the presence of a magnetic field gradient, the droplet moves faster and deforms drastically near the magnet. As the droplet size decreases, the deformation and droplet velocity reduce due to the surface tension effect. By studying the non-uniform magnetic field, they found that magnetic and inertial forces have an essential effect on droplet deformation compared to the uniform magnetic field. However, the droplet elongates in the magnetic field direction similar to the uniform magnetic field. In addition, some studies focused on the deformation of a single falling ferrofluid droplet under gravity in uniform^32^ and non-uniform magnetic fields^33^.

Numerical methods used in droplet deformation have also been utilized in ferrofluid droplet formation. Droplet formation from a linearly magnetizable ferrofluid under a uniform magnetic field is studied numerically and experimentally in a T-junction droplet generator^34,35^. Magnetic force, in their design, elongates the growing tip of the ferrofluid and results in a larger thread and formation time. Ray et al.^36^ used an external magnetic field after a T-junction droplet generator to manipulate the ferrofluid droplets' size by merging them. Using this method, they were able to increase the diameter of the droplets up to 3 times only by tuning the magnetic field intensity. Varma et al.^37^ has also studied ferrofluid droplets generated in a T-junction under a uniform magnetic field. They showed that droplet shape, velocity, size, and inter droplet spacing could be controlled by flow rates, ferrofluid magnetic susceptibility, and continuous-phase viscosity. The effect of uniform magnetic fields on the droplet formation in a co-flowing microchannel is investigated numerically using hybrid lattice Boltzmann and finite volume method by Ghaderi et al.^38^. They confirmed that by increasing magnetic Bond number, the formation frequency and the length of droplet increase whereas the inter droplet spacing decreases. Also, the same pattern is achieved with increasing magnetic susceptibility. Other studies focused on magnetic and hydrodynamic force balance to predict ferrofluid droplet trajectories. These studies assume droplets as magnetic point dipoles^39,40^. Therefore, the effects of droplet deformation are neglected.

Droplet generation by passive methods produces continuous droplet streams. Droplet size and frequency are not tunable in real-time and cannot be controlled independently. Therefore, on-demand droplet formation has been proposed as a solution. The wireless control of on-demand droplet generation by magnetic field has the potential to be used in industrial applications like 3d-printing, inkjet printing, and biomedicine. Precise control of the droplet size and frequency and monodispersity is very crucial in these applications and is dependent on many different parameters. A higher magnetic induction for Drop-on-Demand (DoD) is required compared to the previously mentioned studies in which the droplet size is manipulated by a magnetic field in a continuous droplet stream generated by a syringe pump. Consequently, ferrofluid magnetic susceptibility cannot be assumed constant and show a nonlinear behavior that has not been considered.

In this study, the ferrofluid droplet formation from a nozzle with a non-linear magnetic susceptibility in the presence of a non-uniform magnetic field and gravity is investigated using a multi-scale approach. Studying the dynamics of this phenomenon is interesting due to its rich underlying physics. The interaction of surface tension, gravity, and magnetic force is investigated. The ferrofluid magnetic susceptibility is approximated by the Langevin equation due to high applied magnetic induction values. The gradient of magnetic induction in a non-uniform magnetic field leads to a higher applied force to the ferrofluid relative to a uniform magnetic field. Multi-scale modeling is utilized to incorporate a non-uniform magnetic field effect induced by a relatively large magnetic coil compared to the nozzle size. The ferrofluid droplet generation is investigated experimentally, and the droplet formation process of numerical results is validated against our experimental data. Although the effect of magnetic Bond number has been commonly studied in the literature, gravitational Bond number, Ohnesorge, fluid properties, and geometrical dimensions are left out. Understanding the effect of each parameter could help to improve the performance of the on-demand droplet formation. A comprehensive study is performed to investigate the sensitivity of droplet size and frequency to all dimensionless parameters included in the physics of this problem.

## Mathematical modeling

### Problem description

In this study, ferrofluid droplet formation is investigated numerically and experimentally, and subsequently, the results are compared. Then, the effects of all dimensionless parameters of the problem, which are impossible to study experimentally, are studied numerically. In fact, by changing the material in the experimental method, all the fluid properties, including density, viscosity, and magnetic susceptibility, change simultaneously. Therefore, considering the effect of only one variable without changing others on the droplet diameter and formation frequency is impossible in experimental approaches.

**Figure 1:**
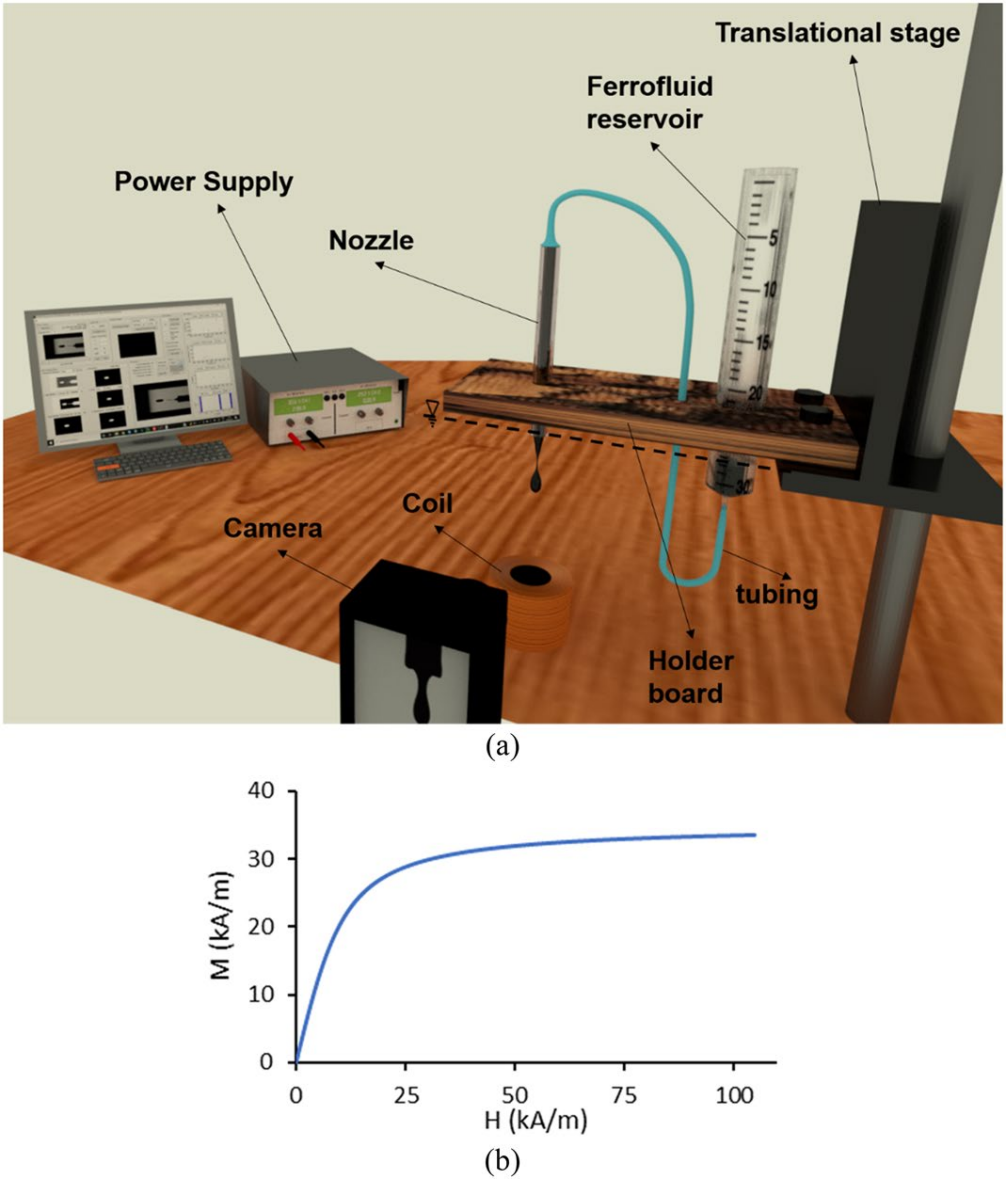
(**a**) Experimental setup, (**b**) magnetization versus magnetic field strength for ferrofluid.

Figure 1a shows the experimental setup. A magnetic coil with a cylindrical ferrite core with a diameter of 2.2 cm and a height of 6.2 cm is used for magnetic field generation (540 rounds of copper wire with a diameter of 1 mm wrapped around a ferrite core with a thickness of 1.75 cm). The strength of the magnetic field generated by the coil can be controlled by an adjustable direct current induced by a DC power supply (MICRO, PW-4053S). The magnetic flux density at the nozzle tip ($B_{N}$) is measured by a gauss meter (MG3002-LUTRON).

The ferrofluid droplet is generated from a glass capillary tube (World Precision Instrument capillary 1B 100-6) with a length of $L_{nozzle}$ = 7.9 cm and inner and outer diameters of $D_{N,i}$ = 0.58 mm and $D_{N}$ = 1 mm. As shown in Fig. 1a, the ferrofluid is connected to a reservoir via a polyethylene tube (Scientific Commodities, Inc) with an inner diameter of $D_{tube}$ = 0.8 mm and length of $L_{tube}$ = 42 cm. Both the capillary tube (nozzle) and reservoir are fixed in a holder board in a way that the tip of the nozzle is at the same height as the ferrofluid-free surface inside the reservoir. In this condition, no ferrofluid droplet is generated in the lack of the magnetic field due to the zero-gauge pressure. Hence, the droplet starts to generate once the magnetic field is turned on. As a result, this setup provides the DoD feature. The ferrofluid inside the reservoir is exposed to the atmosphere, and the reservoir diameter is relatively large compared to that of the nozzle. Hence, the ferrofluid height reduction in the reservoir during the drop formation is negligible, and it can be assumed constant. The reservoir is sufficiently distant from the nozzle, so the magnetic field that arises from the coil has a negligible effect on the ferrofluid inside the reservoir.

The vertical and horizontal positions of the nozzle relative to the magnetic coil can be adjusted by two translational stages (Mahfanavar, one dimensional). In this regard, the nozzle axis and the coil are aligned. Therefore, according to the axisymmetric geometries of the nozzle and cylindrical coil, the problem is solved in two dimensions with the axisymmetric boundary condition. The droplet formation process is recorded by a high-speed camera (Nikon1, J4) with 1200 frames per second, equipped with micro-lens. The subsequent pictures are analyzed by an image processing software^41^ to obtain the diameter and frequency of generated droplets.

An oil-based ferrofluid (EFH1, Ferrotec, USA) is used in experiments consisting of nanoparticles with a nominal diameter of 10 nm in light hydrocarbon oil as a carrier fluid and a volume concentration of 7.9%. The ferrofluid density, viscosity, and initial magnetic susceptibility are equal to $\rho_{f}$ = 1210 kg/m^3^, $\mu_{f}$ = 6 mPa s, and $\chi_{0,f}$ = 2.64, respectively. The saturation magnetization of ferrofluid is 35 kA/m. The surface of the nozzle is hydrophilic, and the contact angle between the ferrofluid and the nozzle surface is measured to be 3° with a surface tension coefficient of 29 mN/m. The magnetization versus magnetic field strength of this ferrofluid is shown in Fig. 1b.

**Figure 2:**
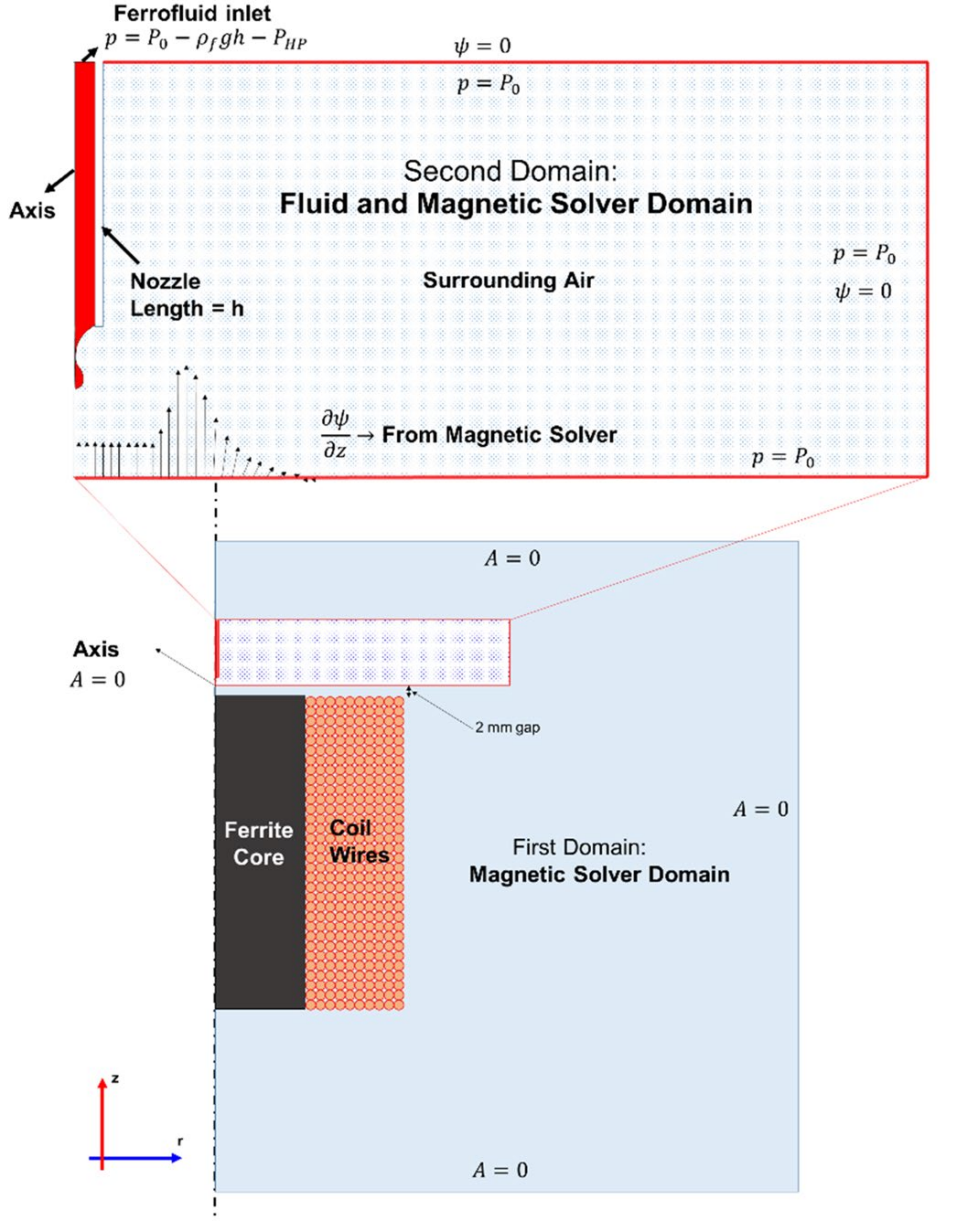
The magnetic and fluid boundary conditions assumed for the two assumed computational domains.

Considering the large dimensions of the magnetic coil relative to the nozzle and the generated drop, it is not possible to consider the coil and its surrounding environment in the computational domain of fluids. A large domain will make the simulation unnecessarily time-consuming and waste computational resources. Therefore, the problem is not solvable in a single computational domain at once; in other words, the computational domain of fluids must be considered small enough to simulate drop formation with high accuracy by numerical methods (multiscale modeling). As shown in the bottom side of Fig. 2, first, a computational domain with dimensions ten times the magnetic coil from the top, right, and bottom of the coil is considered to solve the magnetic equations. This computational domain is shown in light blue as the first computational domain. It is worth mentioning that no fluid flow equations are solved in this domain.

In the next step, after solving the magnetic equations in the first computational domain, a smaller domain as the second computational domain, shown at the top side of Fig. 2, is considered to obtain the drop formation process by solving the Navier–Stokes equations. After solving the fluid flow equations and determining the drop interface in each iteration in this computational domain, the magnetic equation is solved to update the magnetic potential and the magnetic field to calculate the force applied to the drop. Therefore, the coupling of magnetic and fluid flow equations is solved in this computational domain. The magnetic field strength at the lower boundary of the second domain is obtained from the magnetic field solved by the first domain. The lower boundary of the second domain is considered with a distance of 2 mm away from the top surface of the magnetic coil. Therefore, the singularity at the top surface of the coil on the core and coil boundary does not cause an error in the second computational domain. Since the boundaries are sufficiently far from the drop formation region, their effect on the drop is negligible. The axisymmetric boundary condition on the nozzle axis is considered.

To simulate the DoD conditions, the boundary condition $p = P_{0} - \rho_{f} gh - P_{HP}$ is used at the inlet of the ferrofluid into the nozzle. Where h = 2.5 cm is chosen to be a portion of the total length of the nozzle (7.9 cm) in the second computational domain. The important point in this simulation is that the nozzle length is 7.9 cm, and the length of the tube that connects the tank to the nozzle is 42 cm, not all of which can be simulated in the second computational domain. To consider their effect, the flow inside the tube is assumed to be a Hagen–Poiseuille flow after the flow is established, and their pressure difference is calculated with this assumption ${P_{HP} = \frac{{128\mu_{f} Q}}{\pi }\left( {\frac{{L_{tube} }}{{D_{tube}^{4} }} + \frac{{L_{nozzle} - h}}{{D_{N,i}^{4} }}} \right)}$. Where $D_{N,i}$ and $D_{tube}$ are the inner diameters of the nozzle and the tube, which are 580 and 800 µm, respectively. The state that there is no flow in the tube is $P_{HP} = 0$, and the pressure value $P_{0} - \rho_{f} gh$ is assumed to be equal to the atmospheric pressure by considering the static pressure inside the tube. As a result, no drops form when magnetic force is applied to the ferrofluid, and the DoD condition is simulated.

### Governing equations and numerical discretization methods

First, the magnetic field is solved in the larger computational domain by the following equations:

1 $$\nabla .\vec{B} = 0$$

2 $$\nabla \times \vec{H} = \vec{J}$$

3 $$\vec{J} = \sigma \vec{E} + \sigma \vec{V} \times \vec{B} + \overrightarrow {{J_{e} }}$$

where $\vec{B}$ and $\vec{H}$ are the vectors of magnetic flux density and magnetic field strength, respectively. These quantities are proportional ($\vec{B} = \mu \vec{H}$) via magnetic permeability ($\mu$). $\vec{J}$ is total electric current density, $\sigma$ is electrical conductivity, and $\vec{E}$ is the electric field vector. $\left| {\overrightarrow {{J_{e} }} } \right| = \frac{{NI_{coil} }}{a}$, in which $N$ is the number of turns, $I_{coil}$ is the coil current, and $a$ is the cross-section of coil wires shown in Fig. 2. If $\vec{B}$ is considered as a curl of a vector ($\vec{B} = \nabla \times \vec{A}$), in which $\vec{A}$ is the magnetic vector potential, Eq. (1) is satisfied, and Eqs. (2) and (3) can be rearranged into Eq. (4):

4 $$\nabla^{2} A = - \mu \frac{{NI_{coil} }}{a}$$

Note that $\vec{E}$ equals zero in the domain due to net-zero charge, and $A$ is the magnitude of $\vec{A}$ in the tangential direction ($A_{r} = 0$ and $A_{z} = 0$).

As shown in Fig. 2, the larger computational domain consists of three zones, including surrounding air, ferrite core, and coil wires. Equation (4) is solved in each zone by considering the continuity of $A$ on the boundaries between these three zones. $A = 0$ is considered at all boundaries.

Then, to calculate the magnetic field in the smaller domain, the Maxwell equations without any source term must be solved [$\vec{J} = 0$ in Eq. (2)]. In this domain, $\vec{H}$ is considered as a gradient of a potential quantity ($\vec{H} = \nabla \psi$, in which $\psi$ is the magnetic scalar potential), Eq. (2) is satisfied, and Eq. (1) is obtained as follows:

5 $$\nabla .\left( {\mu \nabla \psi } \right) = 0$$

Therefore, Eqs. (1) and (2) can be modified as Eq. (5). Magnetic permeability is a property of a substance that can be denoted by $\mu = \mu_{0} \left( {1 + \chi } \right)$, in which $\chi$ is the magnetic susceptibility. Hence, Eq. (5) becomes:

6 $$\nabla .\left( {\left( {1 + \chi } \right)\nabla \psi } \right) = 0$$

When the magnetic field is calculated, the magnetic force exerted on the ferrofluid can be obtained accurately using the magnetic force ($\vec{F}_{m} = \nabla .{\varvec{\tau}}_{m}$, where ${\varvec{\tau}}_{m} = - \frac{\mu }{2}\left| {\vec{H}} \right|^{2} {\varvec{I}} + \mu \vec{H}\vec{H}^{T}$)^25,26^. However, to reduce the computational cost and complexity, a simpler form of the equation which is valid for linear magnetic susceptibility has been used. $\nabla \chi$ in this equation has been modified by considering nonlinear magnetic permeability through Eqs. (11) to (14). Also, the numerical results obtained by this magnetic force has been validated against experimental data for non-linear magnetic susceptibility in the literature^42,43^.

7 $$\vec{F}_{{\text{m}}} = - \frac{1}{2}\mu_{0} H^{2} \nabla \chi$$

The magnetic force acts only at the regions where the gradient of magnetic susceptibility is nonzero. Calculating the magnetic susceptibility gradient in Eq. (7) requires special considerations because estimating these gradients with a numerical difference between cell values can lead to incorrect calculations at singular points (points on the interface). The magnetic susceptibility changes abruptly across the interface from continuous phase ($\chi_{c}$) to discrete phase $(\chi_{d}$). Therefore, to avoid any discontinuities at the ferrofluid-air interface, the harmonic mean is used for magnetic susceptibility^34,35^:

8 $$\frac{1}{1 + \chi } = \frac{{1 - {\Omega }}}{{1 + x_{c} }} + \frac{{\Omega }}{{1 + \chi_{d} }}$$

where ${\Omega }$ is the Heaviside smooth function, which is a function of the distance from the droplet interface. This function is zero in the continuous phase and one in the discrete phase, and it smoothly increases from zero to one at the boundary by the following equation:

9 $${\Omega } = \left\{ {\begin{array}{*{20}l} {0,} \hfill & {\phi < - \varepsilon } \hfill \\ {\frac{\phi + \varepsilon }{{2\varepsilon }} + \frac{1}{2\pi }\sin \left( {\frac{\pi \phi }{\varepsilon }} \right),} \hfill & {\left| \phi \right| < \varepsilon } \hfill \\ {1,} \hfill & {\phi > + \varepsilon } \hfill \\ \end{array} } \right.$$

In this equation, ε is 1.5 times the size of the smallest mesh in the droplet formation region, and $\phi$ is the level set function, which is obtained from the following equation:

10 $$\frac{\partial \phi }{{\partial t}} + \vec{V}.\nabla \phi = 0$$

The coupling of the level set and VOF methods^44^ is used to solve the two-phase flow.

Although the ferrofluid magnetic susceptibility ($\chi_{d}$) at low magnetic field strength can be assumed constant, it is a function of applied magnetic field strength at high values. As the magnetic field strength increases, the magnetic nanoparticles orient in the direction of the field, so the ferrofluid magnetization becomes saturated, and the magnetic susceptibility decreases. Magnetic susceptibility can be expressed by the Langevin function^42^:

11 $$\chi_{d} = \frac{{M_{s} }}{H}\left[ {\coth \left( {\frac{{3\chi_{0} H}}{{M_{s} }}} \right) - \left( {\frac{{3\chi_{0} H}}{{M_{s} }}} \right)^{ - 1} } \right]$$

where $M_{s}$ and $\chi_{0}$ are the saturation magnetization and initial magnetic susceptibility, respectively. More detail for deriving Eq. (11) could be found in Supplementary information. Although the ferrofluid consists of magnetic nanoparticles with surfactant in a carrier fluid, in the numerical method the ferrofluid is assumed as a uniform material. Chain formation and phase separation may occur under a strong magnetic field^26^. However, these effects are taken into account by considering the Langevin equation for the experimental value of ferrofluid magnetic susceptibility. The magnetic susceptibility in Eq. (11) depends on H and makes a nonlinear system. Therefore, $\chi_{d}$ is calculated explicitly based on the magnetic field strength in the previous time step.

By rearranging Eq. (8), the following equation is obtained:

12 $$\chi = \frac{1}{{\frac{{1 - {\Omega }}}{{1 + \chi_{c} }} + \frac{{\Omega }}{{1 + \chi_{d} }}}} - 1 = \frac{{\chi_{c} \left( {1 + \chi_{d} } \right) + {\Omega }\left( {\chi_{d} - \chi_{c} } \right)}}{{1 + \chi_{d} - {\Omega }\left( {\chi_{d} - \chi_{c} } \right)}}$$

Using the chain rule and assuming that the air as a continuous phase is non-magnetic ($\chi _{c}$ = 0), the magnetic susceptibility gradient becomes $\nabla \chi = \frac{\partial \chi }{{\partial {\Omega }}} \times \frac{{d{\Omega }}}{d\phi }\nabla \phi + \frac{\partial \chi }{{\partial \chi_{d} }} \times \frac{{d\chi_{d} }}{dH}\nabla H$. By rearranging this equation, the magnetic susceptibility is calculated via Eq. (13).

13 $$\nabla \chi = \frac{{\chi_{d} \left( {1 + \chi_{d} } \right)}}{{\left( {1 + \chi_{d} - {\Omega }\chi_{d} } \right)^{2} }}\frac{{d{\Omega }}}{d\phi }\nabla \phi + \frac{{\Omega }}{{\left( {1 + \chi_{d} - {\Omega }\chi_{d} } \right)^{2} }}\frac{{d\chi_{d} }}{dH}\nabla H$$

In the first term of Eq. (13), $\nabla \phi$ is zero everywhere except when $\left| \phi \right| < \varepsilon$.$\frac{{d{\Omega }}}{d\phi }$ and $\frac{{d\chi_{d} }}{dH}$ are obtained using Eqs. (9) and (11), respectively. If the applied magnetic field is uniform and the magnetic susceptibility is constant (in small applied magnetic field strength), then the second term in Eq. (13) becomes zero, and the magnetic force is applied only on the droplet interface. However, in the case of non-uniform magnetic fields, if a strong magnetic field is applied (the magnetic susceptibility is not constant and obeys the Langevin functions), the second term of Eq. (13) results in a body force on the ferrofluid droplet.

$\nabla \phi$ and $\nabla H$ are obtained after solving the fields $\phi$ and $H$, respectively. Considering $\coth \left( {\frac{{3\chi_{0} H}}{{M_{s} }}} \right) = \alpha$ to simplify the equations, the following equation is obtained for calculating $\frac{{d\chi_{d} }}{dH}$ based on Eq. (11):

14 $$\frac{{d\chi_{d} }}{dH} = - \frac{{3\chi_{0} }}{H}\left( {1 - \alpha^{2} } \right) + \alpha \left( { - \frac{{M_{s} }}{{H^{2} }}} \right) + \frac{{2M_{s}^{2} }}{{3\chi_{0} H^{3} }}$$

Navier–Stokes equations are used to solve the flow in both discrete and continuous phases:

15 $$\frac{\partial }{\partial t}\left( {{\uprho }\vec{V}} \right) + \nabla .\left( {{\uprho }\vec{V}\vec{V}} \right) = - \nabla {\text{p}} + \nabla .\left[ {\mu \left( {\nabla \vec{V} + \nabla \vec{V}^{T} } \right)} \right] + \vec{F}_{s} + \vec{F}_{{\text{m}}} + \vec{F}_{g}$$

where the gravity is applied by $\vec{F}_{g}$ and $\vec{F}_{s}$ is the surface tension force and is calculated as follows:

16 $$\vec{F}_{s} = - \sigma k\hat{n}_{f} D\left( \phi \right)$$

where $k$ is the curvature, $\hat{n}_{f}$ is the vector perpendicular to the boundary^45^, and $D\left( \phi \right)$ is a delta function that is 0 except near the interface of the droplet and is defined as follows.

17 $$D\left( \phi \right) = \left\{ {\begin{array}{*{20}l} {\frac{{1 + \cos \left( {\frac{\pi \phi }{\varepsilon }} \right)}}{2\varepsilon },} \hfill & {\left| \phi \right| < \varepsilon } \hfill \\ {0,} \hfill & {otherwise} \hfill \\ \end{array} } \right.$$

Delta function is a derivative of the ${\Omega }$ function ($\frac{{d{\Omega }}}{d\phi }$ in Eq. (13)). In addition, the continuity equation is solved by the following equation:

18 $$\frac{{\partial {\uprho }}}{\partial t} + \nabla .\left( {{\uprho }\vec{V}} \right) = 0$$

The normal magnetic potential gradient ($\frac{\partial \psi }{{\partial z}}$) assigned to the bottom boundary condition of the small computational domain is derived from the solution of the large computational domain ($H_{z} = - \frac{1}{{\mu_{0} }}\frac{\partial A}{{\partial r}}$). Also, the magnetic scalar potential at the upper and right boundaries of the small computational domain is set to zero, which is sufficiently far from the coil. The atmospheric pressure boundary condition is used for all boundaries containing air, and the velocity is extrapolated from the interior in these boundaries.

Governing equations including continuity, momentum, and magnetic equations were solved using a finite-volume-based CFD package. The pressure–velocity coupling has been done using the SIMPLE method^46^. The pressure gradient, momentum, and two-phase VOF-level set equations are calculated by first-order upwind, second-order upwind, and Geo-Reconstruct methods^47^, respectively. The magnetic equations (Eqs. (4) and (6)) are solved by first-order upwind discretization.

### Dimensionless parameters

The numerical analysis in this study investigates the effect of the parameters affecting the diameter and formation frequency of droplet formation. For this purpose, first, all 16 parameters involved in this problem are determined as follows:

- Applied forces: magnetic flux density at the nozzle tip (${B}_{N}$), surface tension ($\sigma$), contact angle ($\theta$), gravitational acceleration (g)
- Geometric parameters of the nozzle and magnetic coil: inner diameter of the nozzle (${D}_{N,i}$), the outer diameter of the nozzle (${D}_{N}$), ferrite core diameter (${D}_{core}$), the winding thickness of the magnetic coil (${t}_{coil}$), the distance between the nozzle and the center of the upper surface of the coil ($L$)
- Properties of ferrofluid and air as the continuous phase: density of ferrofluid (${\rho }_{f}$), the viscosity of ferrofluid (${\mu }_{f}$), initial magnetic susceptibility of ferrofluid ($\chi_{0,f}$), the saturation magnetization of ferrofluid ($M_{s,f}$), density ($\rho_{c}$) and viscosity ($\mu_{c}$) of the continuous phase, and vacuum magnetic permeability coefficient ($\mu_{0}$).

Using Buckingham π theorem and considering the four main dimensions (mass, length, time, and electric charge), the number of parameters can be reduced to 12 dimensionless numbers as follows:

- Force ratios: magnetic Bond number (${\text{Bo}}_{{\text{m}}} = \frac{{B_{N}^{2} L}}{{2\sigma \mu_{0} }}$), gravitational Bond number (${\text{Bo}}_{{\text{g}}} = \frac{{\left( {\rho_{f} - \rho_{c} } \right)gD_{N}^{2} }}{\sigma }$), Ohnesorge number (${\text{Oh}} = \frac{{\sqrt {{\text{We}}} }}{{{\text{Re}}}} = \frac{{\mu_{f} }}{{\sqrt {\rho_{f} \sigma D_{N} } }}$)
- Geometric parameters ratio: nozzle’s inner to the outer diameter ratio ($\frac{{D_{N,i} }}{{D_{N} }}$), the ratio of the distance between the nozzle and the center of the top surface of the coil to the outer diameter of the nozzle ($\frac{L}{{D_{N} }}$), the ratio of ferrite core diameter to outer nozzle diameter ($\frac{{D_{core} }}{{D_{N} }}$), and the ratio of the winding thickness of the magnetic coil to the outer diameter of the nozzle ($\frac{{t_{ coil} }}{{D_{N} }}$) and the contact angle ($\theta$).
- Ferrofluid to the continuous phase properties ratio: dimensionless saturation magnetization (${\overline{\text{M}}}_{{{\text{s}},{\text{f}}}} = M_{s,f} \sqrt {\frac{{\mu_{0} D_{N} }}{\sigma }}$), initial magnetic susceptibility of ferrofluid ($\chi_{0, f}$), viscosity ratio ($\frac{{\mu_{c} }}{{\mu_{f} }}$) and density ratio ($\frac{{\rho_{c} }}{{\rho_{f} }}$).

Also, the dimensionless droplet diameter and formation frequency are expressed by $\frac{d}{{D_{N} }}$ and $F_{f} = \frac{f}{{\sqrt {\frac{\sigma }{{\rho_{f} D_{N}^{3} }}} }}$, respectively.

## Numerical model validation

First, the numerical results are verified with a benchmark. Then, they are validated with experimental results of the ferrofluid droplets formation from the nozzle.

### Verification with a benchmark

A well-known benchmark^48^ is opted to verify the solution results of both magnetic and two-phase flow equations. In this problem, a drop of ferrofluid is suspended in an environment with a uniform magnetic field. Due to the applied magnetic force, the drop begins to stretch until it reaches its equilibrium state. Hence, the drop transfigures from a sphere to an elliptical shape in the direction of the magnetic field. In fact, at this stage, the magnetic field and the surface tension are balanced.

As the magnetic field strength ($H$) increases, the magnetic force applied on the droplet increases, resulting in more elongation of the droplet, which is expressed by shape factor, e, (the ratio of the major to the minor axis) at equilibrium state. In this verification process, a two-dimensional domain with an axisymmetric boundary condition is utilized to simulate the 3D droplet in the benchmark^48^.

**Figure 3:**
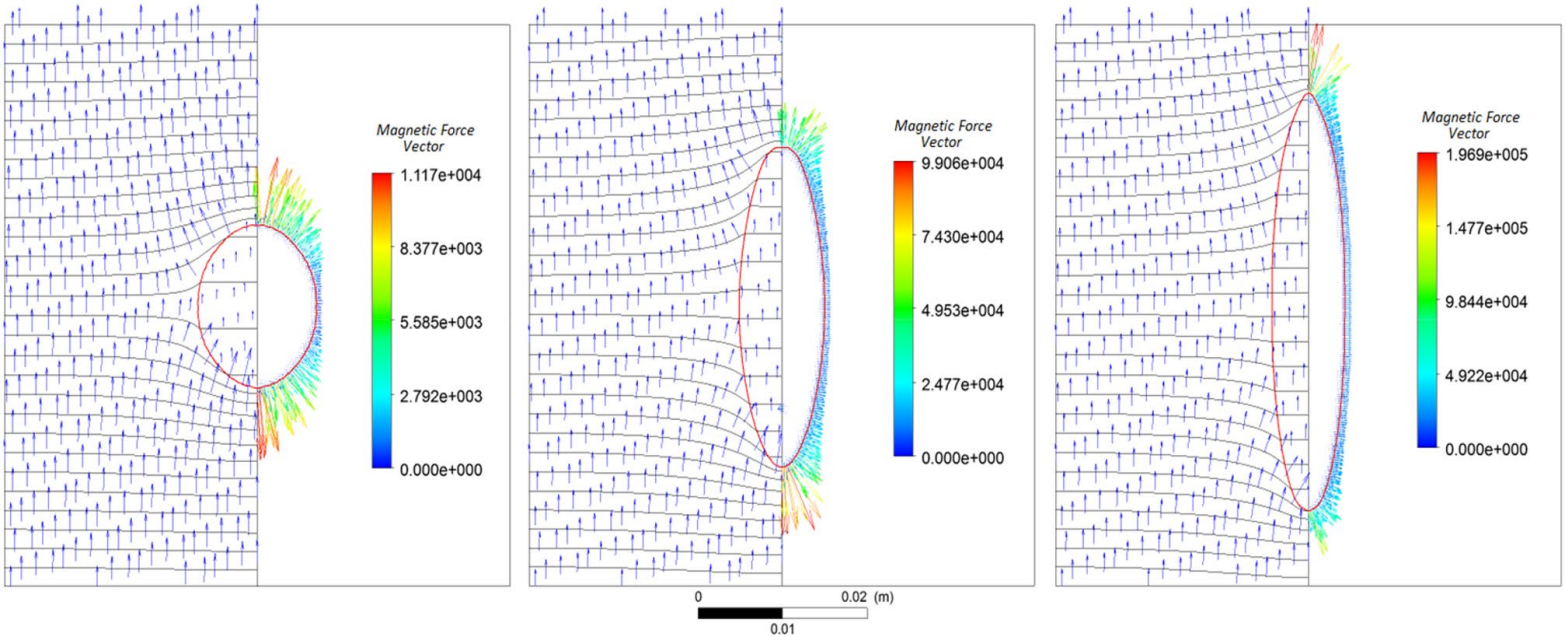
The final shape of the droplet in the steady-state from left to right for three magnetic field strengths of 837, 2043, and 2641 A/m, respectively. In each figure, on the right-hand side, the force vectors with a color map showing the magnetic force magnitude in N/m^3^, and on the left-hand side, the magnetic field vectors with constant magnetic potential lines are shown.

A ferrofluid droplet with a diameter of 1.5 cm, magnetic susceptibility of 5, and a surface tension of 26.5 mN/m is exposed to a uniform magnetic field. The final shapes of the drop in the steady-state condition for the three magnetic field strengths of 837, 2043, and 2641 A/m are shown in Fig. 3 from left to right, respectively. In this figure, the red line shows the droplet interface. On the right-hand side of each part, the applied magnetic force vectors are indicated with a color map, which shows the magnetic force magnitude in N/m^3^. On the left-hand side, the magnetic field vectors (in blue) are shown along with the constant magnetic potential lines (in black), and the red line shows the droplet interface.

It is shown that the constant magnetic field lines are affected in the presence of a ferrofluid droplet due to its magnetic susceptibility, while it is illustrated that the magnetic force applies to the droplet only at the interface. Also, with increasing magnetic field strength, the droplet elongation increases.

**Figure 4:**
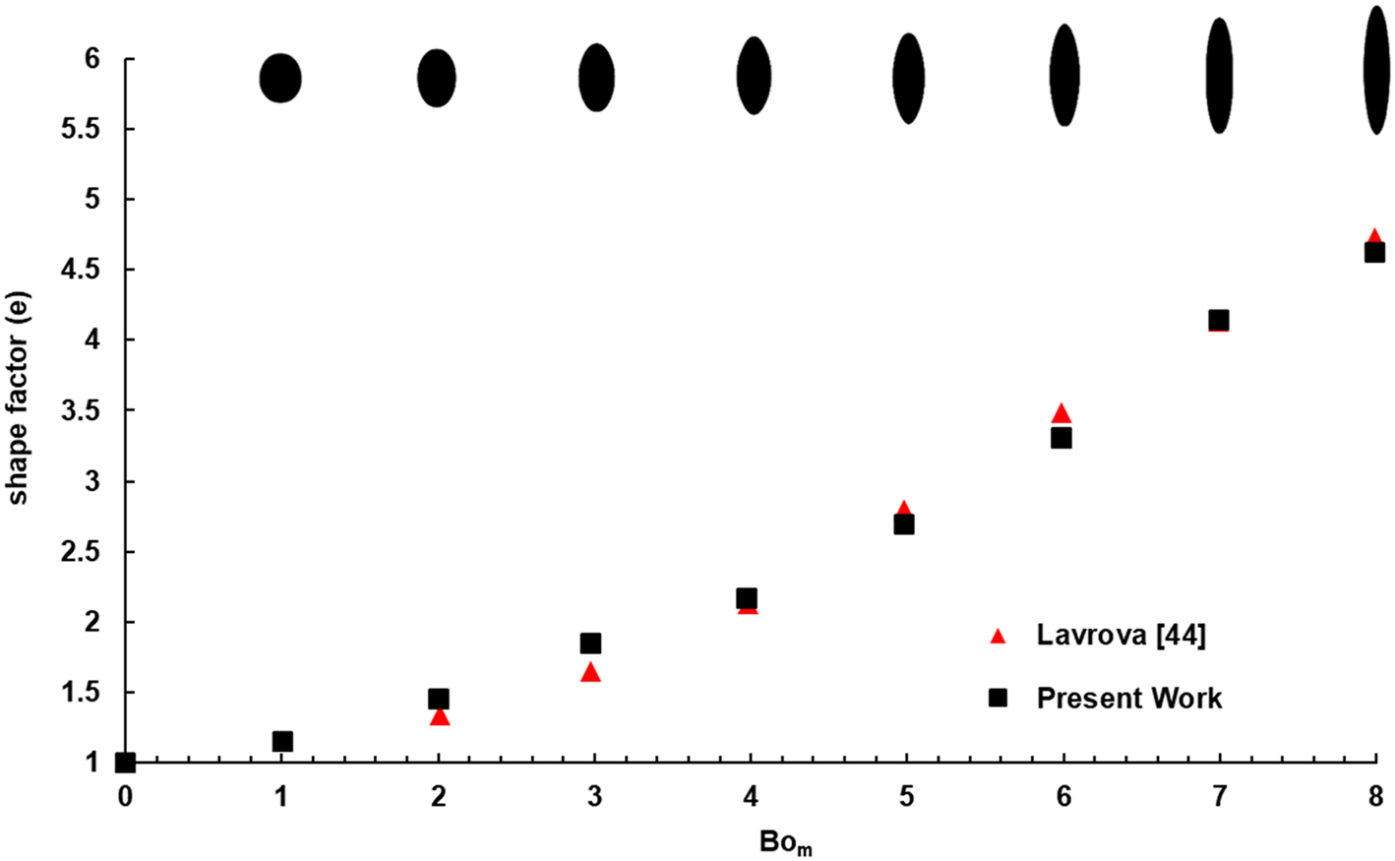
Variations of shape factor (the ratio of the major to the minor axis), e, in terms of the magnetic Bond number using the present numerical method and Lavrova results^48^. The shapes of the droplet in the equilibrium state are shown using the present numerical solution for different magnetic Bond numbers.

Figure 4 illustrates the variation of shape factor (e) in terms of magnetic Bond number (${\text{Bo}}_{{\text{m}}} = \frac{{\mu_{0} V^{1/3} \chi H_{0}^{2} }}{2\sigma }$) in the range of 1 to 8, obtained by the present numerical method and compared with those reported by Lavrova^48^. The shapes of the droplets at the equilibrium state are also shown in this figure using the present numerical solution for different magnetic Bond numbers. Bo_m_ indicates the ratio of magnetic to the surface tension force, and as it increases, the magnetic force can more easily overcome the surface tension. As a result, the ferrofluid droplet is more stretched, and the ratio e increases. As shown, the present results are in good agreement with those of the benchmark. Numerical results have a maximum relative error of 10% occurring Bo_m_ = 3.

### Validation with experimental results and mesh independency

**Figure 5:**
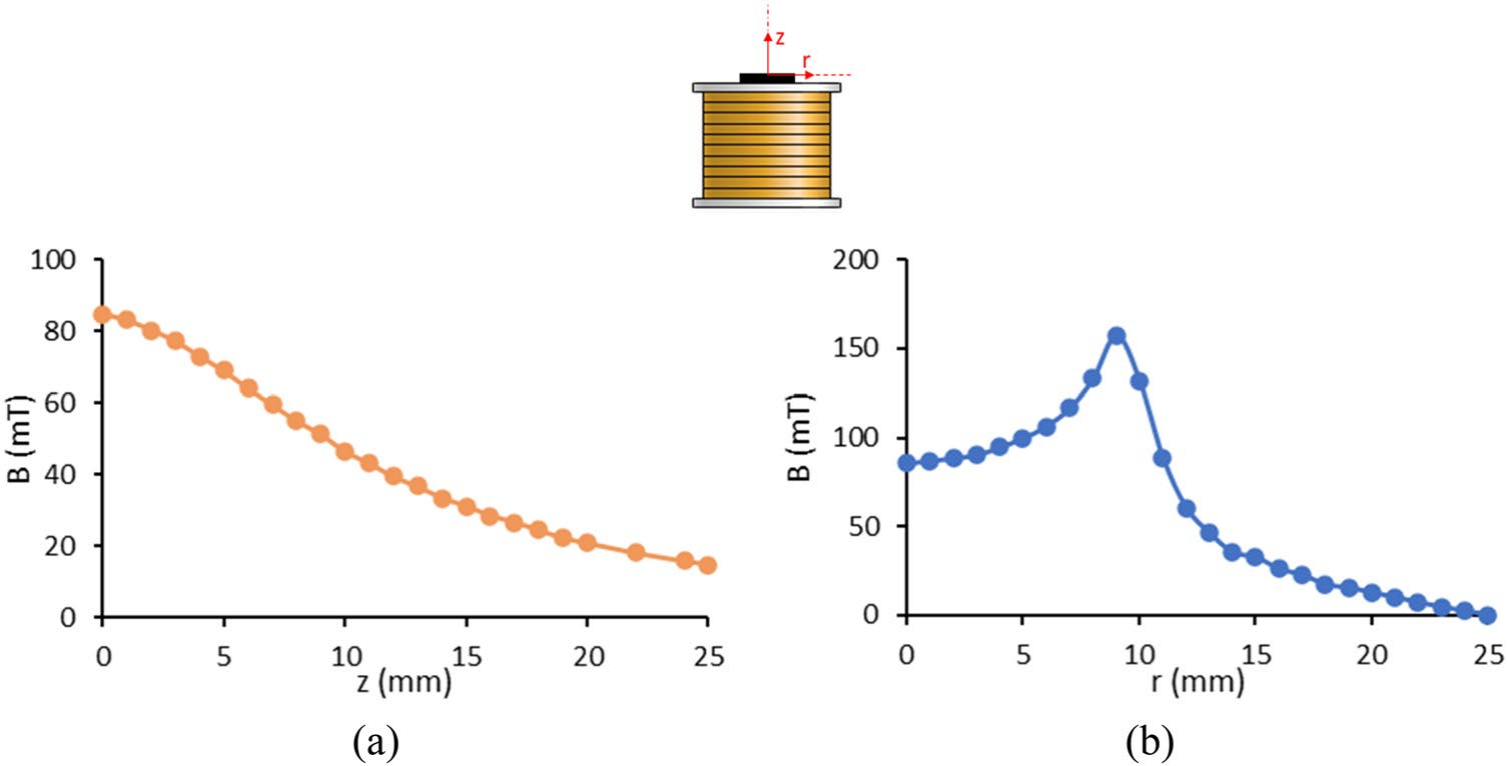
Magnetic flux density in terms of (**a**) the vertical distance on the axis of the magnetic coil and (**b**) the radial distance on the top surface of the magnetic coil.

In this study, the ferrofluid droplet formation is experimentally investigated to validate the numerical method. For this purpose, a current of 3.85 A is applied to the magnetic coil, which creates a magnetic flux density of 58.9 mT at the nozzle tip. The distance between the nozzle and the center of the top surface of the coil is 8 mm. Experimental results of the magnetic flux density on the axis and the top surface of the coil are depicted in Fig. 5a,b, respectively.

**Figure 6:**
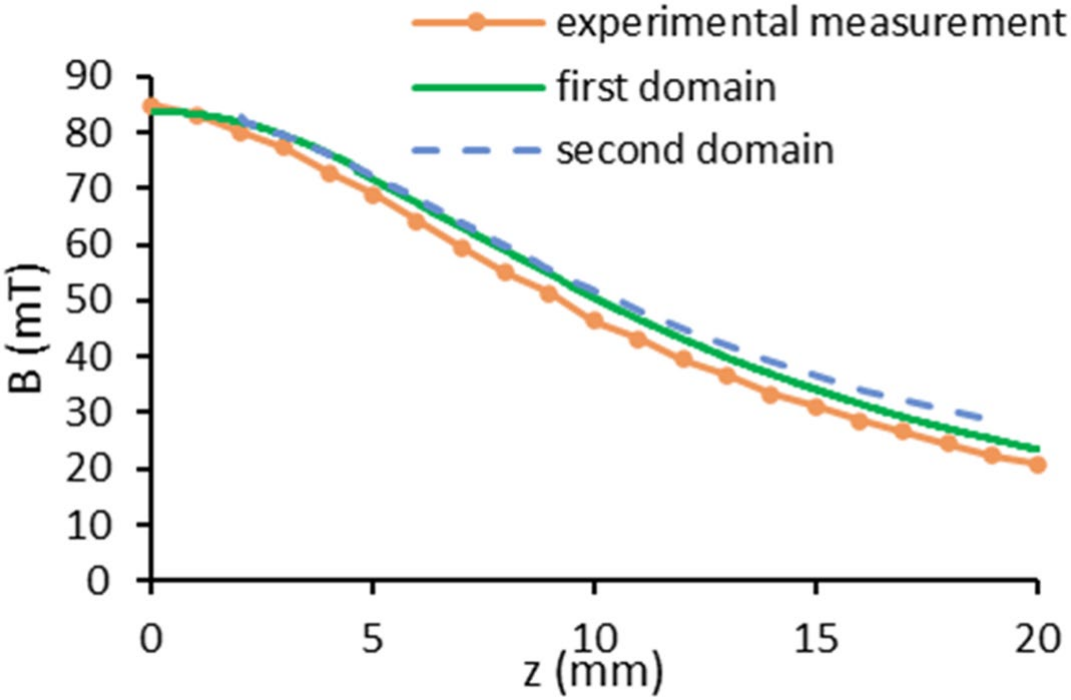
The comparison between the magnetic flux densities of the experimental measurement and the first and second computational domains on the coil axis in terms of vertical distance from the top surface of the magnetic coil.

The magnetic field simulation around the magnetic coil is performed in the first computational domain (magnetic domain) shown in Fig. 2. The results are validated by those illustrated in Fig. 5. Then, as mentioned before, the magnetic induction 2 mm away from the magnetic coil surface obtained from the first domain is assigned to the lower boundary condition of the second domain. Accordingly, solving the magnetic field in the second domain, the values on the coil axis are compared with those of the first domain and the experimental data. Figure 6 shows that there is a good agreement between these three results.

The droplet diameter and time of the first generated droplet are ignored in the numerical simulation due to their dependence on the initial values. Then, the process of the second droplet formation is reported. In fact, the diameter of the second droplet is considered as diameter of the generated droplet and the time interval between the formation of the first and second droplets is considered as the period. Mesh-independency is investigated for six cases with 16,290, 20,080, 24,320, 55,383, 66,992, and 257,757 cells. Finally, the case with 55,383 cells is selected for the calculation having an error of less than 2% compared to the smallest mesh size (maximum number of cells). The minimum grid size, in this case, is equal to 0.015 mm. Also, the ratio of nozzle diameter to minimum grid size is 38. In addition, the Courant number is chosen to be 1 in the simulations.

**Figure 7:**
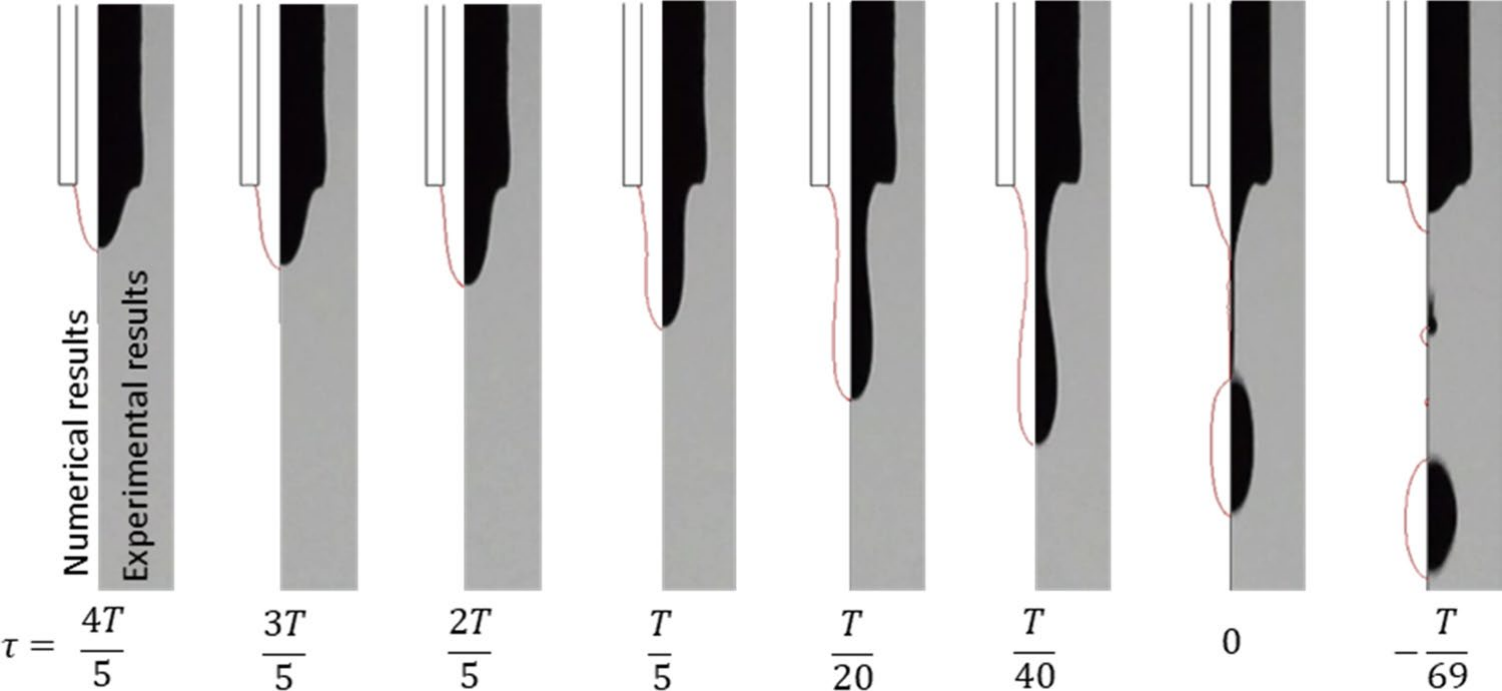
The process of ferrofluid droplet formation under a magnetic field obtained by numerical (left side) and experimental (right side) methods with Bo_m_ = 380.9 and L = 8 mm. $\tau = t_{breakup} - t$ represents the remaining time until the droplet formation, which is expressed in terms of the time period (T) of droplet formation.

Figure 7 shows the process of ferrofluid droplet formation, in which the right and left sides of each part are the experimental and numerical results. The results are presented for L = 8 mm and the magnetic Bond number (${\text{Bo}}_{{\text{m}}} = \frac{{B_{N}^{2} L}}{{2\sigma \mu_{0} }}$) of 380.9. As can be seen, the numerical results for the process of droplet formation are validated by that of the experimental data. In this figure, $\tau = t_{breakup} - t$ is the remaining time until droplet formation, which is expressed in terms of period (T) of droplet formation for the experimental and numerical methods. $\tau = 0$ represents the moment of droplet formation. As can be seen, in both cases the pendant ferrofluid is attracted toward the magnetic coil until the droplet pinch-off. The emergence of necking has started from $\tau = \frac{T}{5}$ until the droplet breakup in both experimental and numerical methods. In addition, the variation of the pendant ferrofluid height versus time is almost identical for both cases, and the rate of this variation increases dramatically near droplet breakup. In this figure, using the numerical method, the diameter and period of the generated droplet are calculated as 797.3 μm and 0.14 s, respectively. However, those of the experimental are measured to be 801.8 μm and 0.17 s, respectively. It is observed that the numerical method can predict the droplet volume of the experiment with an acceptable relative error of 1.7%. Also, the generation of satellite droplet is predicted by numerical method. The relative error of 15.9% is obtained for the time period, in which the error arises from the magnetic field effect on the ferrofluid properties such as viscosity, density, and magnetic permeability at the nozzle tip. However, in the numerical model, the properties of ferrofluid droplets are assumed to be constant and the nanoparticles are considered to be evenly distributed. This condition leads to a delay in droplet formation in the experimental setup compared to the numerical method.

**Figure 8:**
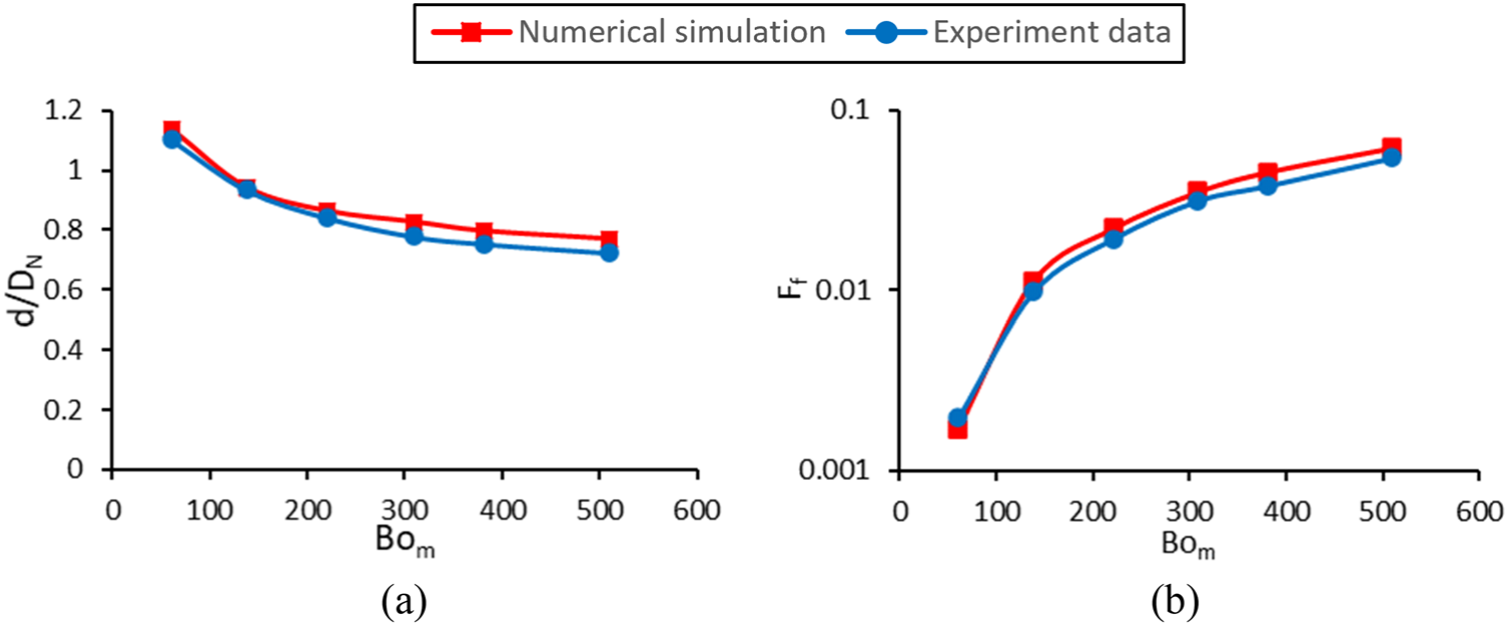
Variations of (**a**) the dimensionless droplet diameter and (**b**) the dimensionless formation frequency in terms of the magnetic Bond number.

In Fig. 7, the validation between numerical and experimental results has been examined in one case with a specific magnetic Bond number. By changing the input current to the magnetic coil, various magnetic flux densities (Bo_m_) are generated at the nozzle tip. The resulting dimensionless droplet diameters and formation frequencies are compared with those of the numerical results in Fig. 8. The numerical results have an average relative error of 13.1% in estimating the droplet volume compared to the experimental data. The average relative error is 14.4% for drop formation frequency. A numerical animation of droplet generation obtained by the present numerical model is found in the supplementary information, Movie S1. In this movie, on the left side, the magnetic force applied to the ferrofluid droplet is shown. On the right side, the deviation of magnetic field lines in the presence of ferrofluid as well as magnetic field strength contour is illustrated.

## Results and discussion

**Figure 9:**
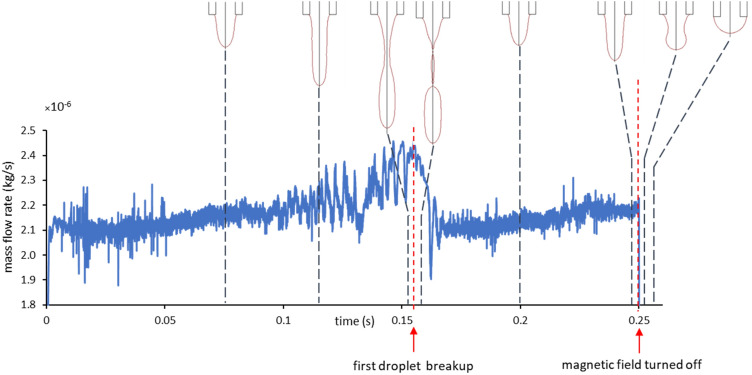
Mass flow rate of ferrofluid from the nozzle versus time showing DoD feature when the magnetic field is turned off at 0.25 s.

To show the possibility of on-demand droplet generation, the magnetic field is turned on and turned off after a time interval equal to 1.7 times of the droplet formation period (t_breakup_ = 0.15 s and t_turn-off_ = 0.25 s). Figure 9 shows the mass flow rate of the ferrofluid from the nozzle during the droplet formation evolution. The forces shown in this figure illustrate that the magnetic and gravitational forces are higher than the surface tension, so the ferrofluid thread is attracted to the magnetic coil, and the flow rate increases until the first droplet is generated. The flow rate during the first droplet generation increases because the magnetic force increase as the volume of pendant ferrofluid increases. An abrupt reduction in the flow rate occurs after the detachment of the first droplet. Again, the mass flow rate increases to generate the second droplet, but once the magnetic field is turned off at 0.25 s, the magnetic force vanishes, and due to the higher magnitude of the surface tension force, the ferrofluid thread retreats to the nozzle, and the flow stops, showing the DoD feature. Consequently, by turning on the magnetic field again, the ferrofluid droplet generation starts. The possibility of on-demand ferrofluid droplet generation by the experimental method is shown in the supplementary information.

In this section, the effect of all 12 dimensionless parameters on the droplet diameter and formation frequency is studied numerically, which cannot be accurately investigated experimentally. For this purpose, dimensionless parameters are examined according to Table 1. These parameters are investigated by 9 cases (case A to I), and corresponding values of fixed dimensionless parameters and the variation range of parameters under investigation are listed in each column. First, the effect of dimensionless numbers representing the ratio of main forces in droplet formation is investigated (case A to C). Then, the effect of geometric variables is studied (case D to F). Finally, the effect of ferrofluid properties is studied (case G to I).

**Table 1:** Selected values and variation intervals of dimensionless numbers.

	Case								
	A	B	C	D	E	F	G	H	I
${\text{Bo}}_{{\text{m}}}$	138.1 to 509.5	138.1 to 509.5	60.7 to 380.9	380.9	220.9	380.9	380.9	380.9	380.9
$\theta$	0.052 to 2.62	0.052	0.052	0.052	0.052	0.052	0.052	0.052	0.052
${\text{Oh}}$	0.024	0.016 to 0.048	0.032	0.032	0.024	0.032	0.032	0.032	0.032
${\text{Bo}}_{{\text{g}}}$	0.24	0.41	0.41 to 15	0.41	0.24	0.41	0.41	0.41	0.41
$\frac{{D_{core} }}{{D_{N} }}$	22	22	22	11 to 44	22	22	22	22	22
$\frac{{t_{coil} }}{{D_{N} }}$	17.75	17.75	17.75	10 to 20	17.75	17.75	17.75	17.75	17.75
$\frac{{D_{N,i} }}{{D_{N} }}$	0.58	0.58	0.58	0.58	0.3 to 0.9	0.58	0.58	0.58	0.58
$\frac{L}{{D_{N} }}$	8	8	8	8	8	8 to 20	8	8	8
$\chi_{0,f}$	2.64	2.64	2.64	2.64	2.64	2.64	0.5 to 4	2.64	2.64
${\overline{\text{M}}}_{{{\text{s}},{\text{f}}}}$	5.55	7.29	7.29	7.29	5.55	7.29	7.29, 20.82	7.29	7.29
$\frac{{\mu_{c} }}{{\mu_{f} }}$	0.00298	0.00298	0.00298	0.00298	0.00298	0.00298	0.00298	0.00298 to 14	0.00298
$\frac{{\rho_{c} }}{{\rho_{f} }}$	0.00101	0.00101	0.00101	0.00101	0.00101	0.00101	0.00101	0.00101	0.00101 to 0.08

### Impact of contact angle (case A)

**Figure 10:**
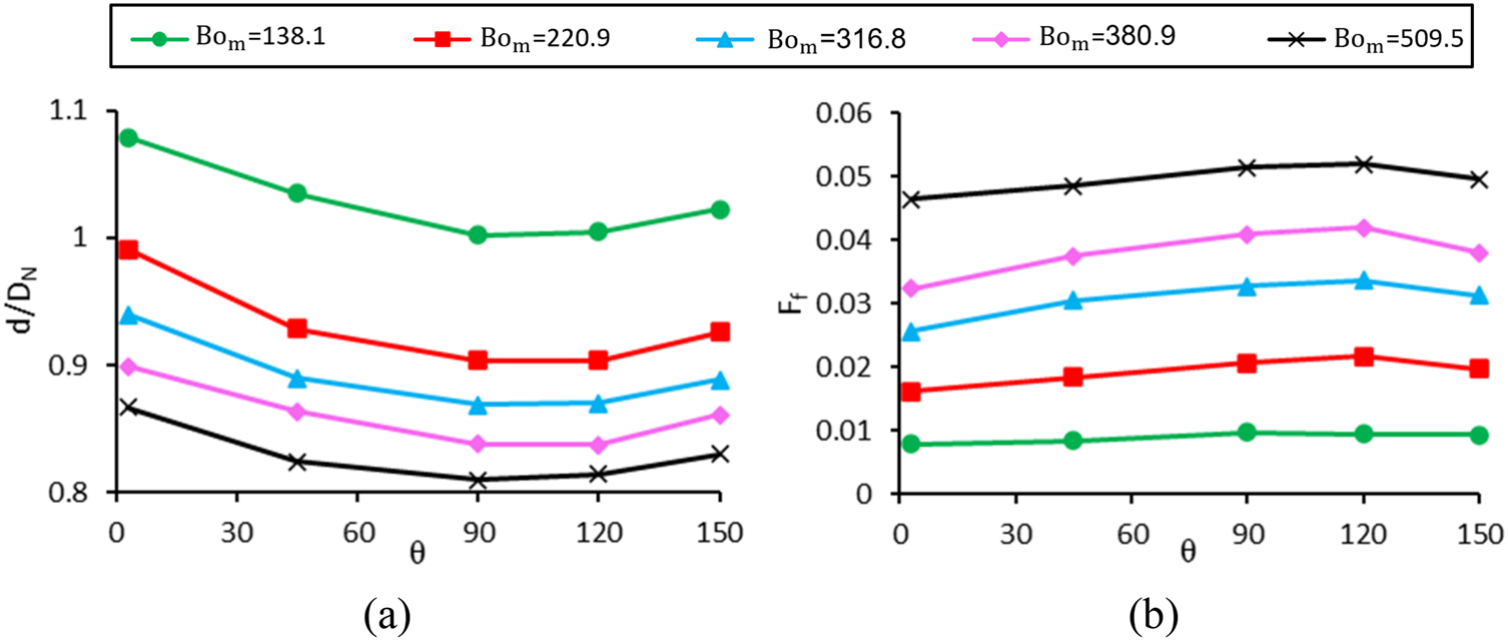
Changes of (**a**) the dimensionless droplet diameter and (**b**) the dimensionless drop formation frequency in terms of contact angle for different magnetic Bond numbers.

The changes in dimensionless diameter and formation frequency in terms of contact angle for different magnetic Bond numbers are investigated in Fig. 10. The values of the fixed dimensionless parameters and the range of variations of the contact angle and the magnetic Bond number are listed in the column determined by case A in Table 1. As reported in the literature^49,50^, the surface tension force increases with increasing the wetted diameter of the nozzle; therefore, by increasing contact angle (nozzle hydrophobicity), the surface tension force decreases. By reducing this force, which resists the formation of the droplet, the droplet becomes smaller and forms faster. As a result, in theory, it is expected that as the contact angle increases, the droplet diameter decreases, and its formation frequency increases. However, as seen in Fig. 10, the diameter of the droplet decreases up to θ = 90° and then increases for θ > 120°. The droplet diameter has a local minimum with respect to contact angle. For a more detailed investigation of this phenomenon, the processes of droplet formation at angles of 3°, 45°, 90° and 120° degrees are shown in Fig. 10 at Bo_m_ = 220.9.

**Figure 11:**
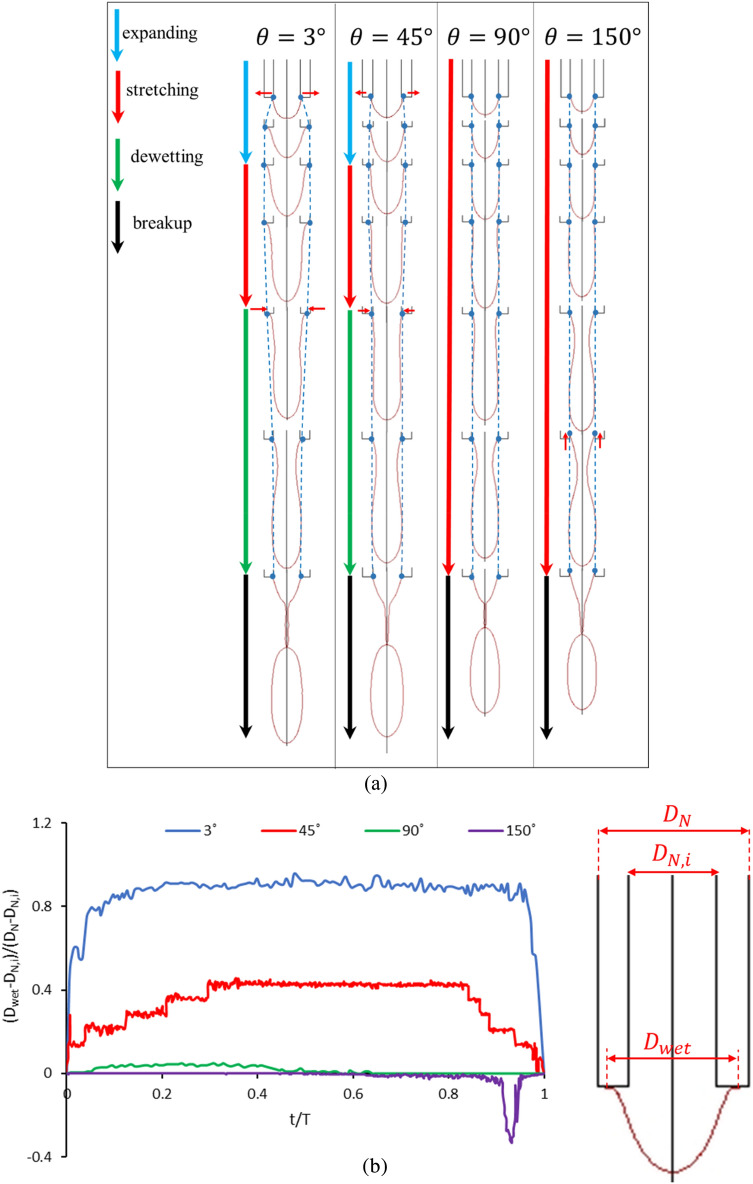
(**a**) Comparison of the droplet formation process and wetted diameter of the nozzle for various contact angles of 3°, 45°, 90°, and 150°. The four mentioned stages are only valid for the contact angles of 3° and 45°. Ferrofluid contact points with the nozzle’s surface are depicted by blue points, and the blue dashed lines show the process of expanding and dewetting. (**b**) Instantaneous dimensionless wetted diameter for contact angles of 3°, 45°, 90°, and 150°.

As shown in Fig. 11a, four stages are observed in the process of drop formation for the contact angle of 3° (hydrophilic nozzle). In the first stage (expanding), ferrofluid grows in the radial direction due to the hydrophilicity of the nozzle and wets the surface of the nozzle. At this stage, the surface tension force dominates the magnetic force. It is worth noting that in this case, the gravitational Bond number is very low, and the gravity can be neglected compared to surface tension and magnetic forces. It is observed that the ferrofluid entirely wets the outer diameter of the nozzle (blue dashed line) due to the small contact angle. In the next stage (stretching), with a further increase in the volume of the suspended ferrofluid, the magnetic force increases and becomes of the same order of magnitude as the surface tension force. Meanwhile, the surface tension holds the suspended ferrofluid at the maximum wetted diameter (outer nozzle diameter at the exit), the magnetic force pulls the ferrofluid towards the magnetic coil. At this stage, the ferrofluid does not grow or shrink in the nozzle’s radial direction while being pulled in the axial direction. Then, the magnetic force dominates the surface tension force (dewetting stage). As the ferrofluid is pulled axially towards the coil, it dewets the nozzle's outer surface (Ferrofluid contact point moves from the outer diameter of the nozzle towards the inner diameter at the exit). Eventually, due to surface instabilities, necking occurs in the elongated thread, and the droplet pinches off (breakup stage). During the ferrofluid droplet generation, the instantaneous wetted diameter ($D_{wet}$) is calculated using the numerical method and the result is presented in Fig. 11b. It is illustrated that for the contact angle of 3°, the wetted diameter starts from the inner nozzle diameter ($D_{N,i}$) and rapidly increases to reach its maximum (expanding stage). Then, the wetted diameter remains at its maximum value for a long-time-interval (stretching stage) until it promptly decreases at the dewetting stage.

The same process is observed in the drop formation process for θ = 45°, and the ferrofluid grows in the radial direction during expanding stage. As the hydrophobicity in the case of the θ = 45° is greater than that of θ = 3°, the ferrofluid does not wet up to the nozzle's outer diameter, and the maximum wetted diameter falls within the inner and outer diameters of the nozzle (marked with a blue dashed line) at the exit. The ferrofluid then grows axially as the wetted diameter remains constant, and after a while, the wetted contact point diameter begins to decrease until a drop is generated. Due to the reduction in the maximum wetted diameter at θ = 45°, a drop is formed with a smaller volume and higher formation frequency than those of θ = 3°. In Fig. 11b, the wetted droplet diameter versus time for contact angle of 45° is shown. Similar to the contact angle of 3°, the wetted diameter increases, remains constant, and finally decreases in the expanding, stretching, and dewetting stages, respectively. The maximum wetted diameter for θ = 45° is smaller than that of θ = 3°.

For θ = 90°, ferrofluid can no longer wet the nozzle thickness at the exit. Hence, the four mentioned stages (expanding, stretching, dewetting, and breakup) observed at θ = 3° and 45° are not seen for this contact angle. Therefore, the expansion and dewetting steps are eliminated, and the drop is pulled axially towards the magnetic coil (stretching) until a drop is generated (breakup). At this contact angle, since the ferrofluid does not wet the nozzle thickness at all, the drop diameter decreases relative to that of θ = 45°, and the formation frequency increases. As shown in Fig. 11b, the wetted diameter remains almost constant and equal to the inner nozzle diameter.

For θ = 150°, the same as 90°, the ferrofluid does not wet the nozzle thickness and is only pulled axially towards the magnetic coil. However, interestingly, it is observed that the wetted surface position )the suspended ferrofluid contact point( moves upward inside the nozzle before the droplet formation occurs due to the high hydrophobicity of the nozzle surface at θ = 150°. Hence, some fluid inside the nozzle is added to the volume of the generated droplet. That results in a larger droplet at the contact angle of 150° than that of 90°. Figure 11b shows that the wetted diameter for θ = 150° is equal to the inner nozzle diameter but at the final stage before the droplet breakup, the ferrofluid moves upward inside the nozzle and the dimensionless wetted diameter becomes negative (the wetted diameter becomes smaller than the inner diameter).

### The effect of Ohnesorge number (case B)

**Figure 12:**
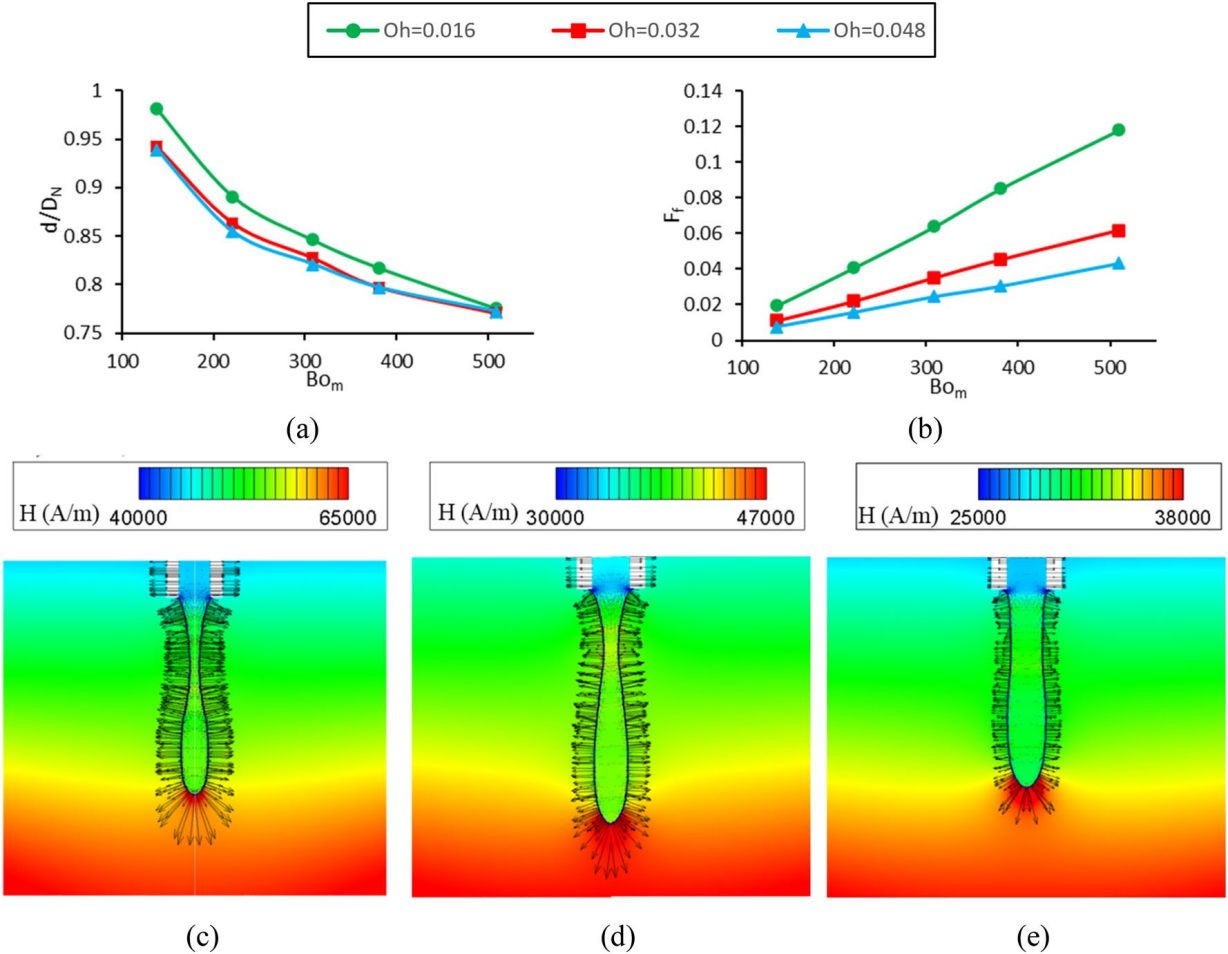
Changes in (**a**) the dimensionless diameter of the drop and (**b**) the dimensionless drop formation frequency in terms of the magnetic Bond number and various Ohnesorge numbers. Magnetic field contours (rainbow color) and vectors of magnetic force (black) for (**c**) Bo_m_ = 380.9, (**d**) Bo_m_ = 220.9, (**e**) Bo_m_ = 138. By decreasing Bo_m_, larger droplets are generated. Note that the maximum values in the color map of magnetic field contours are (**c**) 65,000 A/m, (**d**) 47,000 A/m, (**e**) 38,000 A/m.

The effect of magnetic Bond number on the dimensionless droplet diameter and formation frequency for different Ohnesorge numbers (the ratio of viscous force to inertial and surface tension forces) is shown in Fig. 12a,b, respectively. The constant dimensionless numbers used in this section are presented in the column denoted by case B in Table 1. It is observed that with increasing the magnetic Bond number, the magnetic force increases relative to the surface tension force and so, the droplets are generated with a smaller volume and faster rate. Also, it is shown that by tripling the Ohnesorge number from 0.016 to 0.048, the variations of dimensionless droplet diameter are very small (maximum variation is equal to 4.1%). The drag force applied by continuous phase on the droplet is very low against other forces, and even by tripling this force, no significant effect on the diameter of the drop is observed. Also, the viscosity of ferrofluid affects droplet formation frequency due to the flow inside the connecting tube between the nozzle and reservoir. Figure 12b shows that by increasing the Ohnesorge number, the viscosity of the fluid increases compared to other parameters, causing the drop to generate slower and drop formation frequency increases.

The magnetic field strength contours, as well as the magnetic force vectors, are illustrated for three different magnetic Bond numbers in Fig. 12c–e. As shown, with decreasing magnetic Bond number, the maximum magnetic field strength reduces, and the magnetic vectors’ length representing the magnitude of applied force decreases. The figures illustrate that the main magnetic forces are applied to the droplet interface. Also, small forces are exerted on the whole droplet volume due to the Langevin model and are negligible relative to those applied to the interface.

### Effect of gravitational Bond number (case C)

**Figure 13:**
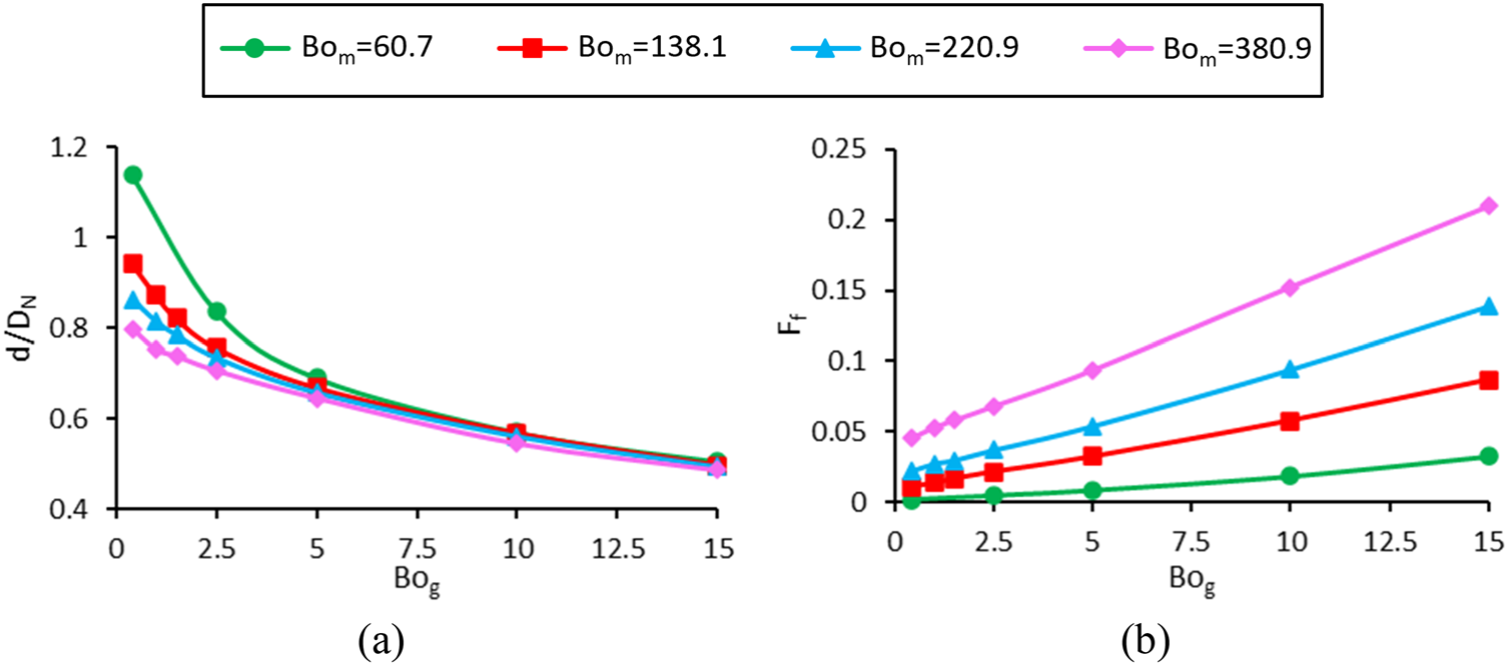
Variations in (**a**) the dimensionless droplet diameter and (**b**) the dimensionless formation frequency in terms of the gravitational Bond number and different magnetic Bond numbers.

In this study, gravitational, surface tension, and magnetic forces are the most important forces in the formation of ferrofluid drops from the nozzle. The ratio of these three forces is expressed by two well-known dimensionless numbers, including the gravitational and magnetic Bond numbers. The simultaneous effect of changes in gravitational and magnetic Bond numbers on the dimensional diameter and its formation frequency is investigated in Fig. 13. The values of the constant dimensionless parameters and the range of variations of the gravitational and magnetic Bond numbers are listed in Table 1, case C.

In the experimental method, independently changing the gravitational Bond number was impossible, and the gravitational force was small compared to the magnetic and surface tension forces. Therefore, the effect of this variable can be investigated by numerical method. It is observed that with increasing the gravitational Bond number, the ratio of gravitational force to surface tension force increases, and the droplet is generated faster with a smaller volume. Figure 13a shows that gravity is predominant at high gravitational Bond numbers (Bo_g_), and the change in the magnetic Bond number (Bo_m_) has little effect on the generated droplet diameter. At low gravitational Bond numbers, the gravitational force decreases and so, the sensitivity of drop diameter to magnetic Bond number increases. For example, at Bo_g_ = 15, increasing Bo_m_ from 60.7 to 380.9, reduces the dimensionless diameter by 3.6%, whereas at Bo_g_ = 0.4, with a similar increase in the Bo_m_, the dimensionless diameter decreases by 30%.

In addition, it is observed that at low magnetic Bond numbers, the sensitivity of the dimensionless droplet diameter to changes in Bo_g_ is higher, and the effect of gravitational force is more pronounced due to the low magnetic force. For example, by decreasing the gravitational Bond number from 15 to 0.4, the dimensionless droplet diameter at Bo_m_ = 60.7 increases by a factor of 2.25, while at Bo_m_ = 380.9, it increases by a factor of 1.64.

### Effect of magnetic coil size relative to nozzle (case D)

In this section, the effect of coil dimensions, including ferrite core diameter and winding area thickness of the magnetic coil on drop diameter and formation frequency, is investigated. The constant dimensionless numbers considered in case D are listed in Table 1. It is worth mentioning that by changing the core diameter of the coil or the thickness of the winding area, the magnetic field strength lines distribution as well as its magnitude at the nozzle tip changes, and so does Bo_m_. To only examine the effect of geometric dimensions of the magnetic coil, the current inside the magnetic coil is adjusted in a way that the magnetic induction at the nozzle tip (and so Bo_m_) remains constant by changing the coil dimensions.

**Figure 14:**
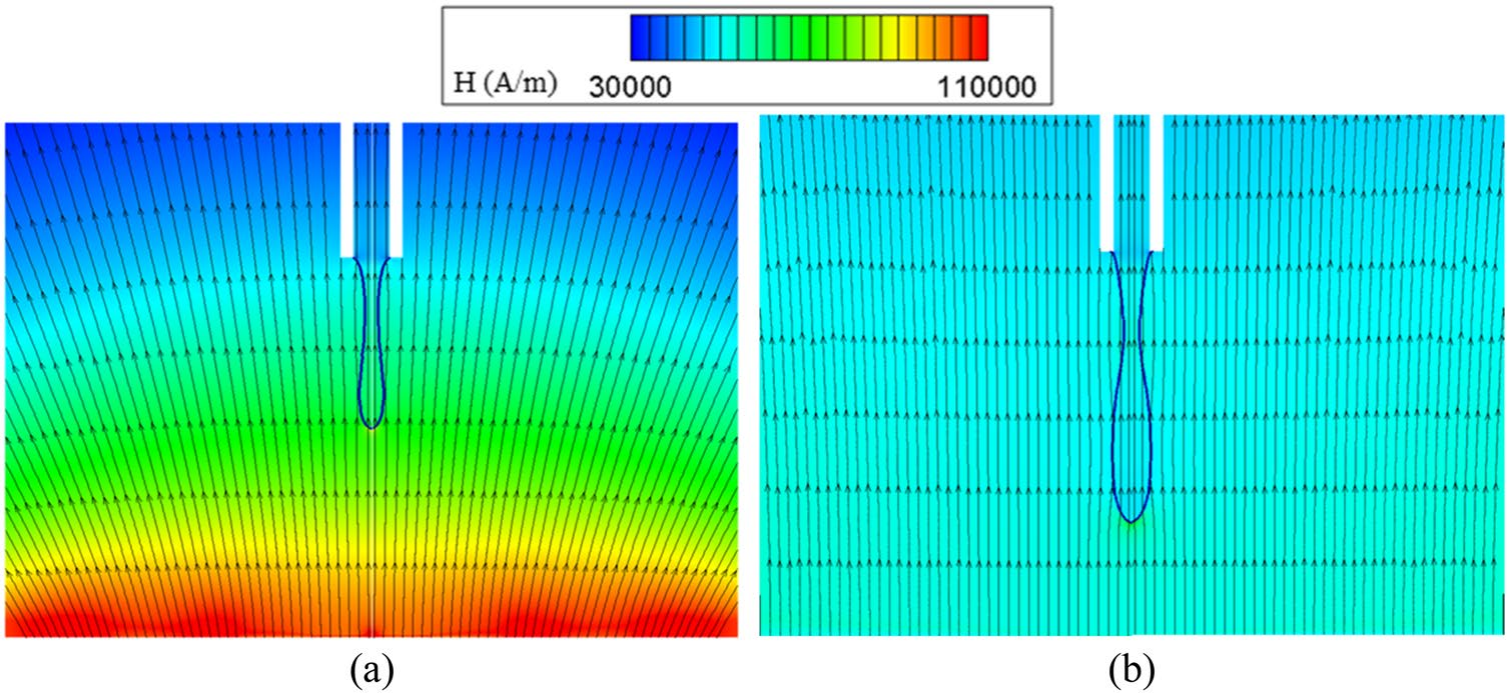
Magnetic field strength contours and magnetic lines for (**a**) $\frac{{D_{core} }}{{D_{N} }} = 11$ and (**b**) $\frac{{D_{core} }}{{D_{N} }} = 44$ for constant $\frac{{t_{core} }}{{D_{N} }} = 17.75$. As the ferrite core diameter increases, the magnetic field around the nozzle tip is more uniform, and the magnetic field lines become parallel.

The effect of ferrite core diameter is investigated in section I of Table 2. Figure 14 shows the magnetic field contours and lines for small and large values of $\frac{{D_{core} }}{{D_{N} }}$ obtained by numerical simulation. As $D_{core}$ increases relative to $D_{N}$, the magnetic field created around the nozzle and the drop formation areas becomes more uniform, and magnetic lines become parallel. Hence, the magnetic field gradient reduces, which leads to a reduction in magnetic force. As the magnetic force decreases, the droplet generates at a slower rate and in a larger volume. Numerical results in Table 2 show that by quadrupling the ratio of the ferrite core to nozzle diameter, the dimensionless droplet diameter increases by 59.3%, and the formation frequency decreases by 83.1%.

**Table 2:**
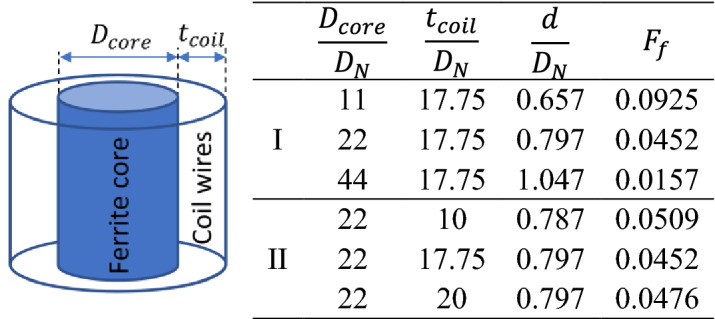
Effect of core diameter and magnetic coil winding area thickness on the dimensionless diameter and formation frequency.

The second section in Table 2 illustrates the effect of the magnetic coil winding area thickness. The magnetic field lines initiate from the ferrite core of the magnetic coil and return to it. So, only the ferrite core size variation has an impact on the magnetic field distribution around the magnetic coil, and the winding area thickness does not affect the magnetic field. Thus, as listed in Table 2, with increasing $t_{coil}$, the drop diameter does not change at all, and the dimensionless formation frequency variation is negligible.

### Effect of nozzle’s inner to outer diameter ratio (case E)

**Figure 15:**
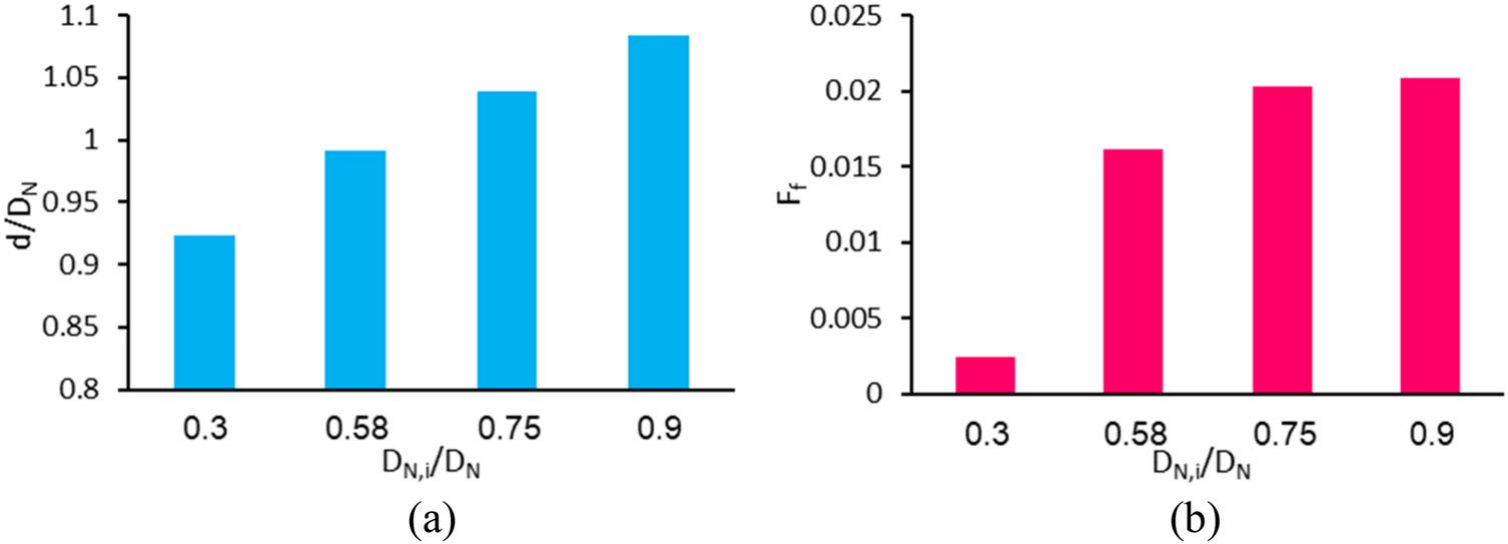
Variations of (**a**) the dimensionless diameter and (**b**) the dimensionless formation frequency in terms of the nozzle’s inner to the outer diameter ratio.

The variations of dimensionless diameter and formation frequency in terms of the nozzle’s inner to outer diameter ratio are shown in Fig. 15. Other dimensionless parameters are assumed constant, as indicated in Table 1 in a column related to case E. It is observed that by reducing the nozzle’s diameter ratio (e.g., by decreasing the nozzle’s inner diameter and assuming a constant nozzle’s outer diameter), the drop diameter decreases. In previous experimental studies, similar dependence of generated droplet relative to nozzle diameter, including the ferrofluid droplet generation in the presence of a magnet^51^ and water drop by injection through a syringe pump^52^, has also been reported. These results show that the generated drop diameter is easily scalable using nozzles and a wide range of drop sizes can be obtained only by changing the nozzle diameter. In all previous studies, by changing some parameters, it was observed that changes in diameter size are inversely related to changes in the formation frequency. However, Fig. 15 shows that as the nozzle’s inner to outer diameter ratio decreases, not only does the generated drop diameter become smaller, but also its formation frequency decreases. In fact, as the nozzle’s inner diameter decreases, the fluid friction inside the nozzle increases. Thus, reducing the nozzle’s inner to outer diameter ratio leads to slower ferrofluid motion in the nozzle and results in a reduction of formation frequency.

### Effect of the ratio of the distance between the nozzle and the center of the top surface of the coil to the outer nozzle diameter (case F)

**Figure 16:**
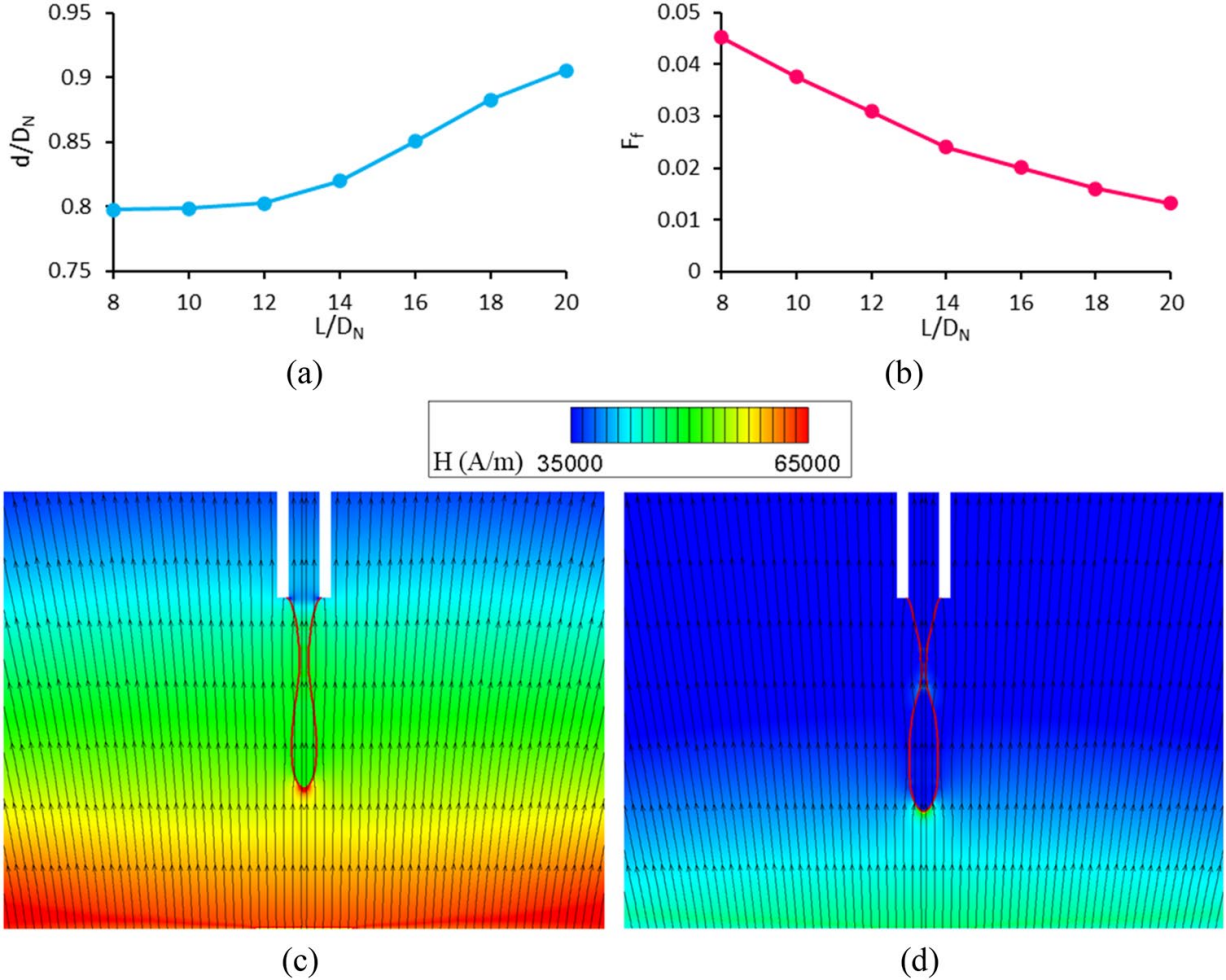
Effect of the ratio of the distance between the nozzle and the center of the top surface of the coil to the outer nozzle diameter on the (**a**) the dimensionless diameter and (**b**) the dimensionless formation frequency. (**c**) Magnetic field strength contours and magnetic lines for (**a**) $\frac{L}{{D_{N} }} = 10$ and (**b**) $\frac{L}{{D_{N} }} = 20$.

In this section, the variation of dimensionless drop diameter and formation frequency are respectively shown in Fig. 16a,b (case F of Table 1). This analysis concerns the ratio of the distance between the nozzle and the center of the top surface of the coil to the outer nozzle diameter. By increasing the distance between the nozzle and coil, the magnetic flux density at the nozzle tip, as well as the magnetic Bond number, decreases. Hence, both the ${\text{Bo}}_{{\text{m}}}$ and $\frac{L}{{D_{N} }}$ change because of a change in L. To only examine the effect of $\frac{L}{{D_{N} }}$, the current inside the magnetic coil is increased to keep ${\text{Bo}}_{{\text{m}}}$ constant while increasing $\frac{L}{{D_{N} }}$. As the distance of the nozzle from the magnetic coil increases, the ferrofluid droplets generate at a further distance from the coil. Therefore, the magnetic field gradient decreases, and the magnetic force reduces. Thus, the ferrofluid droplet is generated with a slower rate (smaller formation frequency) and larger diameter. The magnetic field strength contours and magnetic lines for $\frac{L}{{D_{N} }} = 10$ and $\frac{L}{{D_{N} }} = 20$ are sown in Fig. 16c,d, respectively. It is seen that the magnetic field gradient for a higher distance ratio is smaller.

### Effect of initial magnetic susceptibility and ferrofluid saturation magnetization (case G)

**Figure 17:**
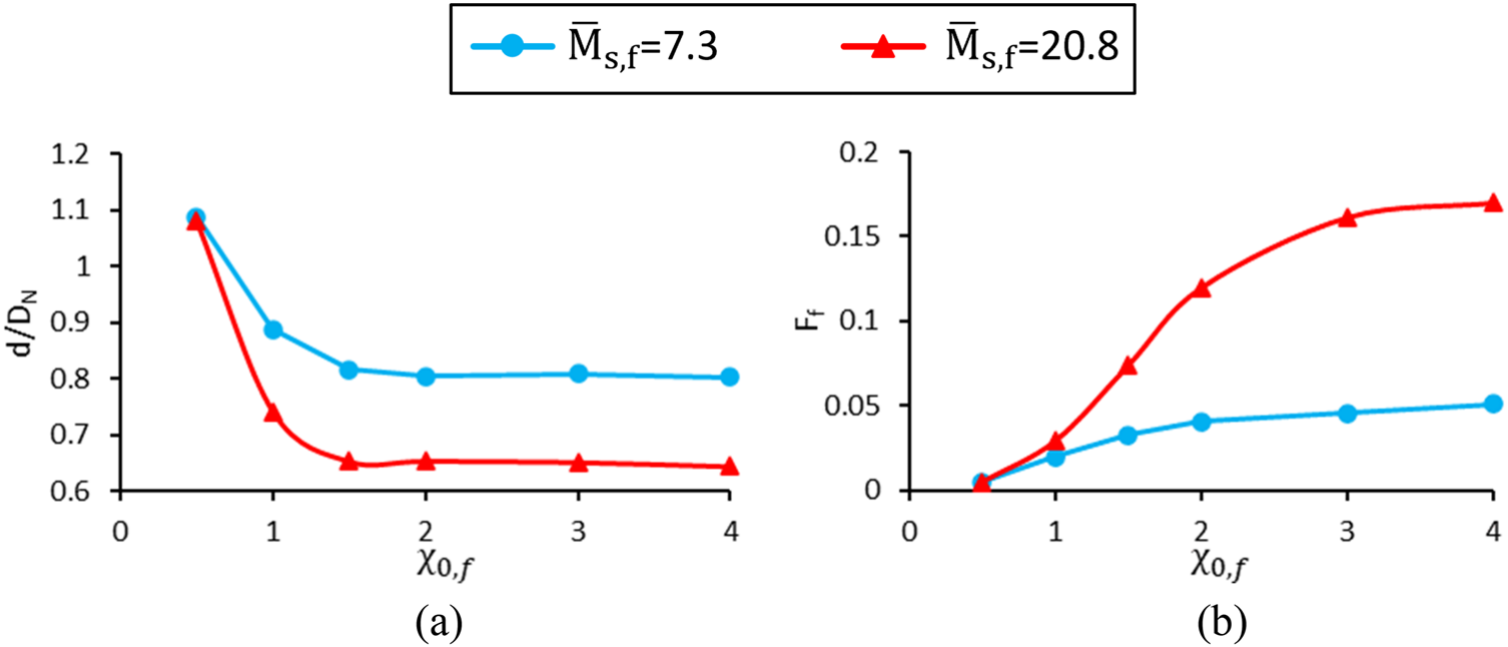
Variation of (**a**) the dimensionless drop diameter and (**b**) dimensionless formation frequency in terms of the initial magnetic susceptibility and various dimensionless saturation magnetizations.

**Figure 18:**
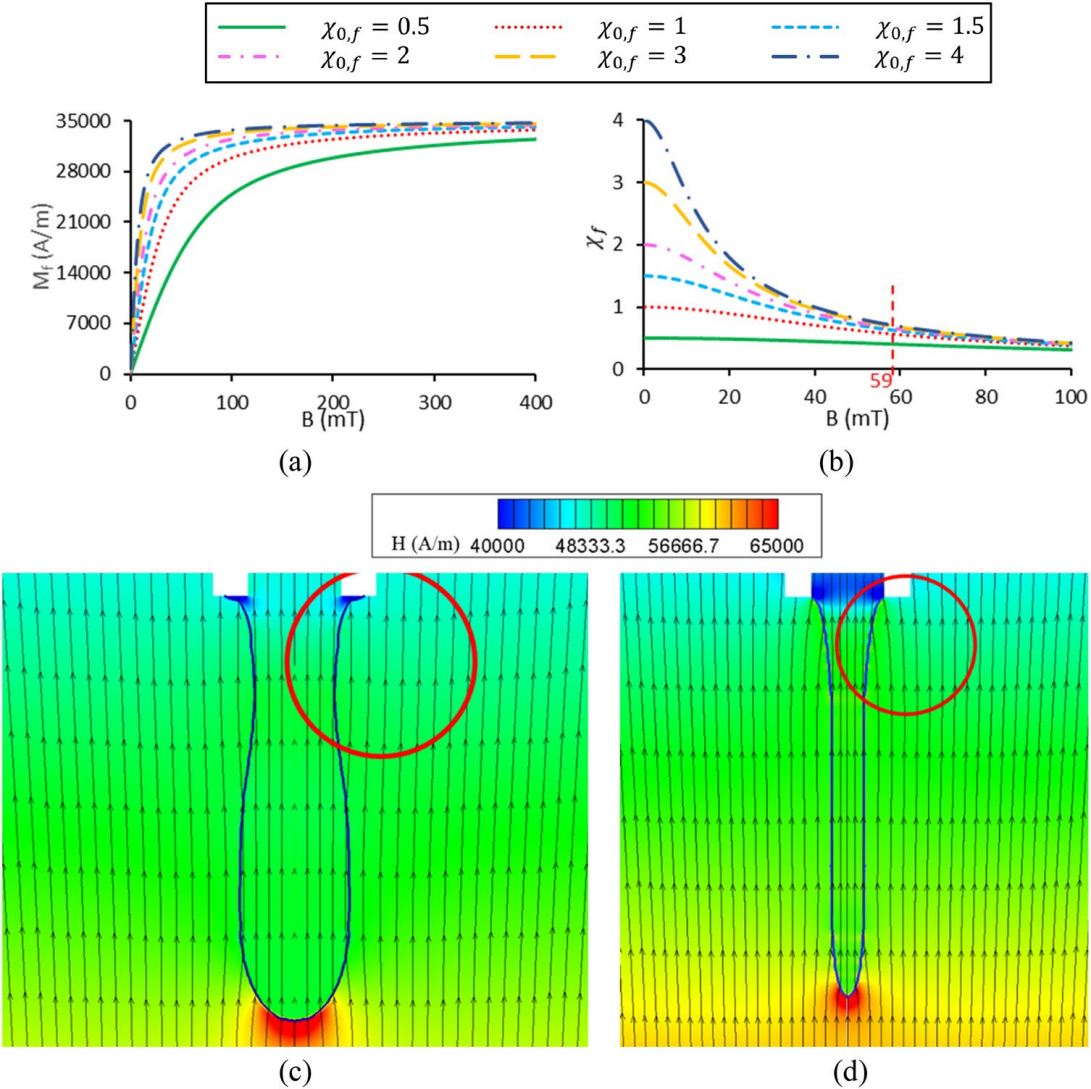
Variations of (**a**) ferrofluid magnetization and (**b**) its magnetic susceptibility in terms of magnetic flux density for various initial magnetic susceptibilities and dimensionless saturation magnetization equal to 7.3. The contours of magnetic field strength and magnetic field lines are obtained numerically for initial magnetic susceptibilities of (**a**) 0.5 and (**b**) 2.

Figure 17 shows the ferrofluid initial magnetic susceptibility and saturation magnetization effects on the dimensionless drop diameter and formation frequency. The other dimensionless parameters are assumed constant and listed in a column related to case G in Table 1. It is observed that with increasing initial magnetic susceptibility ($\chi_{0,f}$) or dimensionless saturation magnetization (${\overline{\text{M}}}_{{{\text{s}},{\text{f}}}}$), the dimensionless drop diameter decreases, whereas its formation frequency increases. As stated in this study, high magnetic flux densities are applied to the ferrofluid. Hence, its magnetic susceptibility cannot be assumed to be constant. Therefore, $\chi_{f}$ is considered to follow the Langevin equation determined by Eq. (11). To understand the physics of this phenomenon, the variations of the ferrofluid magnetization and magnetic susceptibility in terms of magnetic flux density are depicted in Fig. 18a,b, respectively, for various $\chi_{0,f}$ at ${\overline{\text{M}}}_{{{\text{s}},{\text{f}}}} = 7.3$.

Figure 18a illustrates that as the initial magnetic susceptibility increases, the slope of the graph enhances at zero magnetic flux density. The dimensionless saturation magnetization is assumed constant so, all diagrams converge to the same value of saturation magnetization.

The magnetic flux density applied by the magnetic coil at the nozzle tip is 59 mT, which is specified in Fig. 18b. It is observed that with increasing $\chi_{0,f}$, the ferrofluid magnetic susceptibility at this magnetic flux density becomes greater. With increasing magnetic susceptibility, the gradient created at the ferrofluid-air interface enhances, and according to Eq. (7), greater values of magnetic force are obtained. Therefore, the drop is generated faster with a smaller volume. For $\chi_{0,f} = 0.5$ and 2, the magnetic field strength contours and magnetic field lines obtained by the numerical method are illustrated in Fig. 18c,d, respectively. As shown, higher $\chi_{0,f}$ leads to more deviation in magnetic field lines and higher magnetic field gradients resulting in a stronger applied magnetic force. Thereby, for a constant dimensionless saturation magnetization, as the ferrofluid initial magnetic susceptibility increases, the dimensionless diameter of the droplet reduces, whereas the formation frequency increases.

The interesting point that can be seen in Fig. 17a is that the droplet diameter converges to a constant value as $\chi_{0,f}$ increases from 1.5 onwards. For high values of $\chi_{0,f}$, the dimensionless drop diameter converges to 0.81 for ${\overline{\text{M}}}_{{{\text{s}},{\text{f}}}} = 7.3$ and 0.65 for ${\overline{\text{M}}}_{{{\text{s}},{\text{f}}}} = 20.8$. To better understand this issue, the magnetic susceptibility values at the nozzle tip ($B_{N} = 59 mT$) are provided in Table 3, section I. It is observed that by changing $\chi_{0,f}$ from 0.5 to 1.5, the magnetic susceptibility increases by 54.8%, while by elevating $\chi_{0,f}$ from 1.5 to 4, the magnetic susceptibility enhances by only 12.4%. Hence, the variation of $\chi_{f}$ is almost insensitive to changes in $\chi_{0,f}$ for $\chi_{0,f} > 1.5$. Consequently, the dimensionless diameter changes in this interval will be negligible.

**Table 3:** Magnetic susceptibility values at the nozzle tip.

	$\chi_{0,f}$	${\overline{\text{M}}}_{{{\text{s}},{\text{f}}}}$	$\chi_{f}$
I	0.5	7.3	0.402
	1	7.3	0.561
	1.5	7.3	0.623
	2	7.3	0.654
	3	7.3	0.685
	4	7.3	0.700
**II**	**0.5**	**7.3**	**0.402**
	0.5	20.8	0.484
	4	7.3	0.700
	4	20.8	1.754

Significant values are in bold.

**Figure 19:**
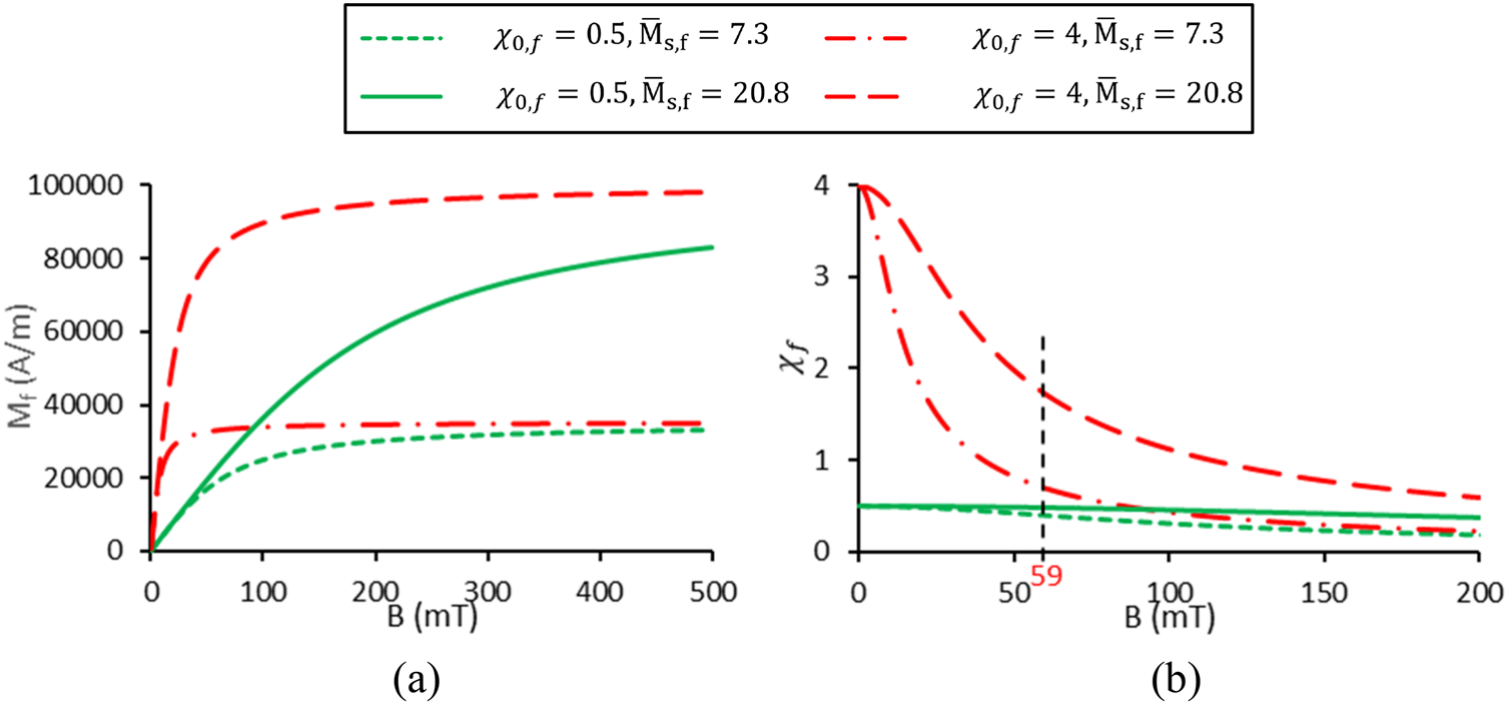
Variations of (**a**) ferrofluid magnetization and (**b**) magnetic susceptibility in terms of magnetic flux density for different initial magnetic sensitivities and dimensionless saturation magnetizations.

To examine the effect of saturation magnetization, the ferrofluid magnetization and the magnetic susceptibility variations in terms of magnetic flux density are shown in Fig. 19a,b, respectively. It is observed that by increasing the saturation magnetization at a constant initial magnetic susceptibility, the magnetization converges to a greater extent, while the slopes of the diagrams at zero magnetic flux density are equal. Figure 19b shows that for a constant magnetic flux density applied to the nozzle tip (59 mT) and a constant $\chi_{0,f}$, the magnetic susceptibility increases with increasing ${\overline{\text{M}}}_{{{\text{s}},{\text{f}}}}$. Thus, $\nabla \chi_{f}$ at the droplet’s interface enhances, and the magnetic force increases. As a result, increasing the ferrofluid dimensionless saturation magnetization leads to a reduction in dimensionless droplet diameter and formation frequency enhancement.

Another interesting point observed in Fig. 17a is that at low values of the initial ferrofluid magnetic susceptibility ($\chi_{0,f} = 0.5$), the droplet diameters for both saturation magnetizations are almost equal. To better understand this issue, the magnetic susceptibility for two saturation magnetizations (${\overline{\text{M}}}_{{{\text{s}},{\text{f}}}} =$ 7.3 and 20.8) and high ($\chi_{0,f} = 0.5$) and low ($\chi_{0,f} = 0.5$) initial magnetic susceptibilities are compared in Fig. 19b.

Figure 19b shows the magnetic susceptibility at the nozzle tip ($B_{N} = 59 mT$), and the values of $\chi_{f}$ are listed in Table 3 (section II). It is observed that at high initial magnetic susceptibility ($\chi_{0,f} = 4$) with increasing dimensionless saturation magnetization, the magnetic susceptibility increases by a factor of 2.5. However, this enhancement is a factor of 1.2 for low initial magnetic susceptibility ($\chi_{0,f} = 0.5$). Thus, considering the changes in magnetic susceptibility, the variation of drop diameter is sensitive to the changes in ${\overline{\text{M}}}_{{{\text{s}},{\text{f}}}}$ at high values of $\chi_{0,f}$. However, as $\chi_{0,f}$ decreases, its sensitivity decreases.

### Effect of continuous phase to ferrofluid viscosity ratio (case H)

**Figure 20:**
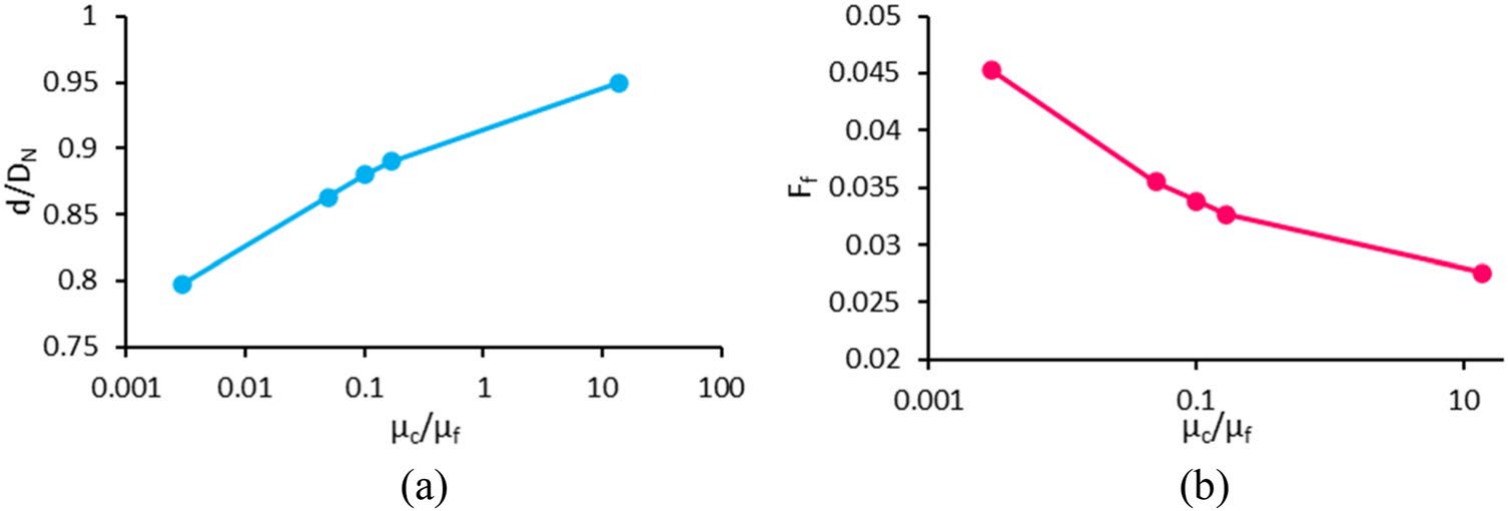
Variation of (**a**) the dimensionless droplet diameter and (**b**) the formation frequency in terms of the continuous phase to ferrofluid viscosity ratio (the x-axis is plotted in a logarithmic scale).

The variations of dimensionless diameter and formation frequency in terms of continuous phase to ferrofluid viscosity ratio are depicted in Fig. 20a, b, respectively. The other dimensionless parameters are considered constant and listed in Table 1 (case H). By increasing continuous phase viscosity relative to ferrofluid viscosity, the magnetic, gravitational, and surface tension forces remain constant, and only the drag force ($F_{D} = \frac{1}{2}\rho_{c} AC_{D} v^{2}$) changes. To understand the physics of this phenomenon, by assuming the droplet as a sphere, the Schiller and Naumann correlation^53^ can be used to obtain the drag coefficient for Re < 1000, as follows:

19 $$C_{D} = \frac{{24\left( {1 + 0.15{\text{Re}}^{0.687} } \right)}}{{{\text{Re}}}}$$

Because the droplet velocity is very low, the condition of Re < 1000 is satisfied, and Eq. (19) is used. The variation of $C_{D}$ relative to ${\text{Re}}$ in this equation is descending, so with increasing the continuous phase viscosity, ${\text{Re}} = \frac{{\rho_{c} vD}}{{\mu_{c} }}$ decreases, and $C_{D}$ increases. Since other parameters in the drag force equation are constant, increasing $C_{D}$ leads to an increase in drag force (which, like surface tension force, resists ferrofluid separation from the nozzle and the formation of droplets). Therefore, the droplets are generated at a slower rate with a larger volume. As a result, by increasing the viscosity ratio of the continuous phase to ferrofluid, the formation frequency decreases, and the droplet diameter increases.

### Effect of continuous phase to ferrofluid density ratio (case I)

**Figure 21:**
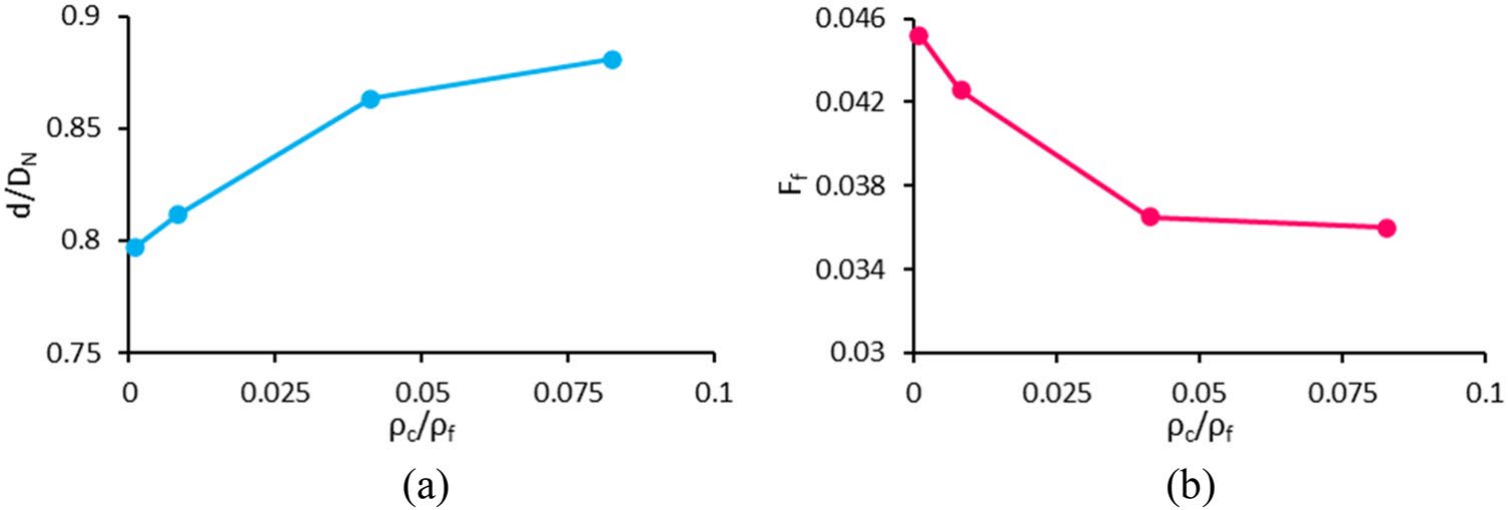
Variation of (**a**) the dimensionless droplet diameter and (**b**) formation frequency versus the continuous phase to ferrofluid density ratio.

Figure 21 shows the effect of continuous phase to ferrofluid density ratio on the dimensionless droplet diameter and its formation frequency. As the density of the continuous phase changes, the density difference and the applied buoyancy force to the drop varies, followed by a change in the gravitational Bond number (${\text{Bo}}_{{\text{g}}} = \frac{{\left( {\rho_{f} - \rho_{c} } \right)gD_{N}^{2} }}{\sigma }$). In the numerical simulations, the gravitational acceleration is set so that the buoyancy force applied to the drop and the gravitational Bond number are kept constant. Hence, only the effect of the density ratio is investigated. The constant parameters assumed in this section are given in the column related to the case I in Table 1. In this case, by changing the density ratio, all forces remain constant except the drag force (similar to the previous section). With increasing continuous phase density, the Reynolds number becomes greater and so, the drag coefficient decreases according to Eq. (19). Although $C_{D}$ decreases with $\rho_{c}$ , the drag force is proportional to the density factor in the drag coefficient ($F_{D} \propto \rho_{c} C_{D}$), and the variation of $F_{D}$ becomes ascending in terms of continuous phase density. Therefore, as the continuous phase density increases, $F_{D}$ becomes stronger. Increasing this force, which resists the formation of droplets, causes larger droplets to be generated at lower frequencies. The experimental study of ferrofluid droplet generation in the air is compared with that of in the water and could be found in the supplementary information.

## Conclusion

In this study, the formation of ferrofluid droplets with a non-linear magnetic susceptibility under a non-uniform magnetic field is numerically and experimentally investigated. Unlike previous studies in which a syringe pump has been used for injecting fluid (constant velocity boundary condition), in this investigation, a zero gradient pressure boundary condition is utilized. Accordingly, the DoD feature is simulated. The problem is solved in two dimensions with an axisymmetric boundary condition. Due to the large size of the magnetic coil relative to the nozzle, the problem is solved by multiscale modeling. First, the Maxwell equations are solved to obtain the magnetic field in a large computational domain around the magnetic coil. Second, the magnetic boundary conditions are assigned to the smaller computational domain using the magnetic field data achieved from the larger domain. The fluid equations (including the continuity, the Navier–Stokes in both discrete and continuous phases, and the two-phase flow equations), as well as the Maxwell equations, are solved for the smaller domain. The finite volume method is utilized for discretization, and coupling of the level set and VOF methods is used to solve the two-phase flow. Magnetic susceptibility is expressed by the Langevin function. In this study, a simpler equation for calculating magnetic field has been used to reduce the complexity and computational cost. However, this equation is modified by considering nonlinear magnetic permeability in the gradient of magnetic susceptibility. Also, numerical results are validated and are in good agreement with a benchmark and experimental data. In the future studies, the upper range of magnetic fields should be explored below which this equation for magnetic force can predict the experimental results, precisely.

The effect of 12 dimensionless parameters on droplet diameter and formation frequency are investigated. The important results can be summarized as follows:

- A minimum in droplet diameter variation versus the contact angle is observed at a contact angle of 90˚ to 120˚.
- As the magnetic and gravitational Bond numbers increase, the magnetic and gravitational forces become greater relative to the surface tension force, respectively, and droplets are generated with a smaller volume and higher formation frequency.
- By increasing the Ohnesorge number, the generated drop diameter variation in the range of studied parameters is negligible because of the low drag force relative to other forces. While, due to the high friction of Poiseuille flow inside the nozzle, the droplet formation frequency decreases.
- As the diameter of the ferrite core increases with respect to the outer nozzle diameter, the magnetic field generated around the nozzle tip and the droplet formation region becomes more uniform, resulting in a reduction of magnetic field gradient and magnetic force. Hence, drops with a larger volume and a lower frequency are generated.
- The winding thickness of the magnetic coil area has no effect on the magnetic field distribution, and its variation does not affect the droplet size and formation frequency.
- As the nozzle’s inner to outer diameter ratio reduces, the droplet diameter and its formation frequency decrease.
- Increasing the distance between the nozzle and the center of the top surface of the coil, makes the magnetic field lines more uniform and parallel. Therefore, both the magnetic field gradient and magnetic force reduces, and the droplet diameter increases, whereas the formation frequency decreases.
- Increasing the initial magnetic susceptibility or the dimensionless saturation magnetization increases the magnetic susceptibility gradient on the ferrofluid interface. Consequently, the magnetic force increases, which leads to a decrease in the diameter of the drop and an increase in its formation frequency.
- By increasing initial magnetic susceptibility, the drop diameter converges to a constant value. In addition, at low initial ferrofluid magnetic susceptibility values, the droplet diameter for different saturation magnetizations almost remains constant.
- As the viscosity and density ratios of the continuous phase to ferrofluid increase, the magnetic, gravitational, and surface tension forces remain constant, while the drag force applied to the suspended ferrofluid becomes greater in value. This fact causes the generation of larger droplets with lower formation frequencies.

## Supplementary Information

Supplementary Information 1.

Supplementary Information 2.

Supplementary Video 1.
