# Supplementary material for: On-demand ferrofluid droplet formation with non-linear magnetic permeability in the presence of high non-uniform magnetic fields
Source: Sci Rep. 2022 Jun 27;12:10868. doi: 10.1038/s41598-022-14624-w (PMC9237107; doi:10.1038/s41598-022-14624-w)
Supplement: Supplementary file 1 — Supplementary Information 1. [file 41598_2022_14624_MOESM1_ESM.pdf]

Supplementary information

On-demand ferrofluid droplet formation with non-linear magnetic permeability in the presence of high non-uniform magnetic fields

Mohamad Ali Bijarchi[a,&], Mohammad Yaghoobi[a,&], Amirhossein Favakeh[a], Mohammad Behshad Shafii[a,*]

[&] These authors contributed equally to this work.

[*] Corresponding author: behshad@sharif.edu

[a] Department of Mechanical Engineering, Sharif University of Technology, Tehran, Iran

A. Langevin equation for the magnetic susceptibility of ferrofluid

The ferrofluid susceptibility could be defined by the Langevin equation. The original Langevin equation for ferrofluid magnetization [1,2] is as follows:

$$M = M_s L(\alpha) = M_s\left(\coth(\alpha) - \frac{1}{\alpha}\right), and\ \alpha = \frac{\pi}{6}\frac{\mu_0 M_d H d^3}{k_B T} \qquad \text{(S1)}$$

Where μ_0 is the vacuum magnetic permeability coefficient, M_d is the domain magnetization, H is the magnetic field strength, d is the nanoparticles diameter, k_B denotes the Boltzmann constant, and T is the temperature. Also, by defining $m = \frac{\pi}{6} M_d d^3$, the Langevin equation is expressed by the following equation [3]:

$$M = M_s L(\alpha) = M_s\left(\coth(\alpha) - \frac{1}{\alpha}\right), and\ \alpha = \frac{\mu_0 m H}{k_B T} \qquad \text{(S2)}$$

The M-H curve of ferrofluid is obtained experimentally and the nanoparticles' diameter and saturation and domain magnetization could be obtained by fitting the above equation to the experimental data. However, in this study, the aim is to investigate the effect of saturation magnetization (M_s) and initial magnetic susceptibility (χ_0) on the ferrofluid droplet formation. Hence, noting the definition of ($\chi_0 = \frac{dM}{dH}|_{H=0}$), by making the derivative from Equation (S2) and using the Taylor series for $\coth^2(\alpha)$, the following equation could be obtained:

$$\chi_0 = \frac{dM}{dH}|_{H=0} = M_s \frac{\mu_0 m}{k_B T}\left(1 - \coth^2(\alpha) + \frac{1}{\alpha^2}\right) = M_s \frac{\mu_0 m}{k_B T} \times \frac{1}{3} \qquad \text{(S3)}$$

By rearranging Equation (S3):

$$\frac{3\chi_0}{M_s} = \frac{\mu_0 m}{k_B T} \qquad \text{(S4)}$$

By substituting Equation (S4) into Equation (S2), the magnetization in terms of saturation magnetization, initial susceptibility, and the magnetic field strength is derived [4, 5]:

$$M = M_s\left(\coth\left(\frac{3\chi_0 H}{M_s}\right) - \left(\frac{3\chi_0 H}{M_s}\right)^{-1}\right) \qquad \text{(S5)}$$

Noting the definition of magnetic susceptibility ($\chi = M/H$), Equation (11) in the text is obtained from Equation (S5).

B. On-demand droplet generation by the experimental method

The possibility of on-demand ferrofluid droplet generation by the experimental method is illustrated in Fig. S1. The ferrofluid is located around the nozzle tip when there is no magnetic field as shown in the left picture. Once the magnetic field is turned on, the ferrofluid is attracted toward the magnetic coil until the first droplet breakup. Then, the ferrofluid moves toward the coil

until the magnetic field is turned off. At this moment, the ferrofluid thread bounces back to the nozzle tip and the process of second droplet generation stops. Hence, the ferrofluid droplet generation can be controlled on-demand by a magnetic field.

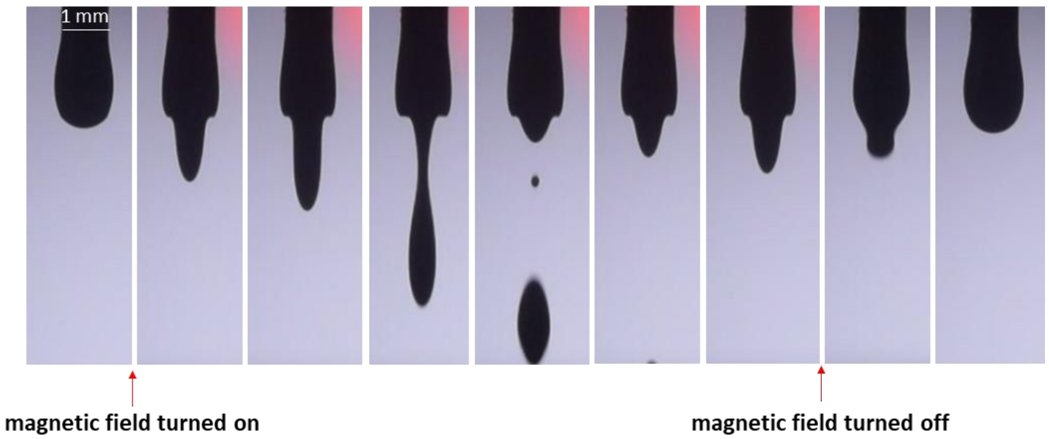

Fig S1. On-demand ferrofluid droplet generation by experimental method. The bright red light in the upper right corner of images shows the moment when the magnetic field is on.

C. The effect of different continuous phases on droplet formation using the experimental method

The ferrofluid droplet formation under a magnetic field in the air is compared with that in the water. As shown in Fig. S2, the dimensionless droplet diameter decreases and formation frequency increases with increasing magnetic Bond number for both cases. In fact, by increasing the magnetic Bond number, the ratio of magnetic force to surface tension increases and leads to a faster generation of smaller droplets. As can be seen, in all magnetic Bond numbers, the droplet diameter generated in the air is smaller than that of in the water. Also, the dimensionless formation frequency of droplets formed in the air is higher than that of in the water. Since the drag force of water as a continuous phase applied to the ferrofluid thread is higher than that of the air, the droplets in the water generate with slower rates and larger diameters.

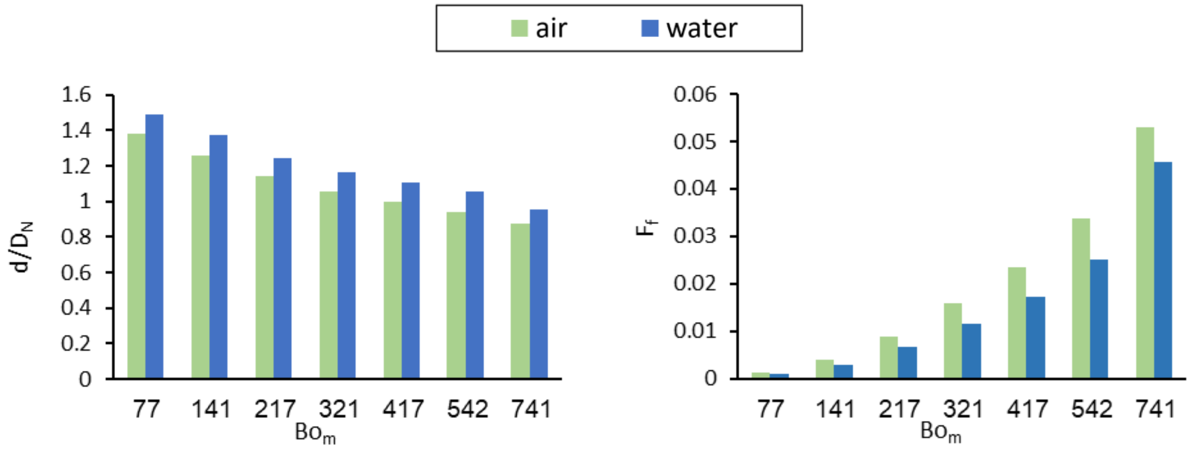

Fig S2. Comparison of ferrofluid droplet formation in air and water as the continuous phase. Variations of (a) the dimensionless droplet diameter and (b) the dimensionless formation frequency in air and water versus the magnetic Bond number.

References

[1] R.E. Rosensweig, Ferrohydrodynamics, Courier Corporation, 2013.

[2] S. Afkhami, Y.Y. Renardy, M.J. Renardy, J.S. Riffle, T. St Pierre, Field-induced motion of ferrofluid droplets through immiscible viscous media, Journal of Fluid Mechanics, (2008).

[3] S.a. Afkhami, A. Tyler, Y.Y. Renardy, M.J. Renardy, T. St Pierre, R. Woodward, J.S. Riffle, Deformation of a hydrophobic ferrofluid droplet suspended in a viscous medium under uniform magnetic fields, Journal of Fluid Mechanics, (2010).

[4] G.-P. Zhu, N.-T. Nguyen, R.V. Ramanujan, X.-Y. Huang, Nonlinear deformation of a ferrofluid droplet in a uniform magnetic field, Langmuir, 27(24) (2011) 14834-14841.

[5] O. Lavrova, Numerical methods for axisymmetric equilibrium magnetic-fluid shapes, Thesis (2006).
