# Supplementary material for: On-demand ferrofluid droplet formation with non-linear magnetic permeability in the presence of high non-uniform magnetic fields
Source: Sci Rep. 2022 Jun 27;12:10868. doi: 10.1038/s41598-022-14624-w (PMC9237107; doi:10.1038/s41598-022-14624-w)
Supplement: Supplementary file 2 — Supplementary Information 2. [file 41598_2022_14624_MOESM2_ESM.docx]

Supplementary of

**On-demand ferrofluid droplet formation with non-linear magnetic permeability in the presence of a non-uniform magnetic field**

Legends:

**Supplementary Movie S1.** Numerical animation of droplet generation obtained by the present numerical model. In this movie, on the left-side the magnetic force applied to the ferrofluid droplet is shown and on the right-side, the deviation of magnetic field lines in the presence of ferrofluid as well as magnetic field strength contour are illustrated.
